# Scientific Mapping of Problem-Based and Project-Based Learning for Environmental Literacy Education: A Bibliometric and Content Analysis

**DOI:** 10.12688/f1000research.182697.1

**Published:** 2026-08-07

**Authors:** Mellyzar Mellyzar, Nahadi Nahadi, Hernani Hernani, Asep Supriatna, Oktariani Oktariani, Marhami Marhami, Desi Aryanti Nabuasa

**Affiliations:** 1 Science Education, Universitas Pendidikan Indonesia, Bandung, West Java, 40154, Indonesia; 2 Chemistry Education, Universitas Pendidikan Indonesia, Bandung, West Java, 40154, Indonesia; 3 Mathematics Education, Universitas Malikussaleh, Lhokseumawe, Aceh, 24351, Indonesia

**Keywords:** Problem-Based Learning, Project-Based Learning, environmental literacy, Education for Sustainable Development, bibliometric analysis, science education

## Abstract

**Background:**

Problem-Based Learning (PBL) and Project-Based Learning (PjBL) have emerged as strategic pedagogies for cultivating environmental literacy (EL) and sustainability competencies within the Education for Sustainable Development (ESD) agenda. Yet the intellectual structure, thematic evolution, and conceptual integration of this body of scholarship remain insufficiently mapped, particularly in relation to the Sustainable Development Goals (SDGs) and to disciplinary contexts such as chemistry and environmental chemistry education.

**Methods:**

This study integrates bibliometric science mapping with systematic content analysis to examine the scholarly landscape of PBL and PjBL-based EL research published in Scopus-indexed journals between 2011 and 2026 (n = 48). Bibliographic data were retrieved through a Boolean query, screened in accordance with the PRISMA 2020 framework, and analyzed using VOSviewer and the Bibliometrix R package to generate co-authorship, keyword co-occurrence, and thematic-evolution maps. A complementary systematic content analysis coded each included study along five dimensions: educational level, learning approach, learning outcomes, SDG alignment, and research methodology.

**Results:**

The field exhibits an accelerating trajectory after 2021, anchored by Indonesian and North American institutional clusters and organized around four thematic clusters: experiential PBL/PjBL pedagogy, sustainability-oriented curriculum reform, empirical literacy assessment, and emerging eco-literacy innovation. Higher education dominates the corpus (48%); SDGs 4, 12, and 13 are the most invoked goals. Persistent gaps include the limited integration of chemistry and environmental chemistry education, weak longitudinal designs, and underdeveloped EL assessment instruments.

**Conclusion:**

PBL and PjBL serve as enabling pedagogical approaches for transformative environmental learning. Future research should prioritize longitudinal designs, validated assessment instruments, interdisciplinary integration across chemistry and other natural sciences, and broader representation from the Global South to strengthen sustainability-oriented science education within the ESD agenda.

## 1. Introduction

The intensification of the planetary ecological crisis manifested in accelerating climate change, biodiversity collapse, freshwater depletion, soil degradation, and the persistent overshoot of multiple planetary boundaries has reframed the moral and intellectual mission of contemporary education ( Leal Filho et al., 2021; Steffen et al., 2018). The United Nations 2030 Agenda for Sustainable Development, and in particular Target 4.7 on ESD, positions formal and informal learning as strategic levers for promoting the cognitive, behavioral, and value-based transformations required for ecological transition ( Rieckmann, 2018). Yet the persistence of environmentally unsustainable practices despite decades of awareness-oriented campaigns has exposed the inadequacy of information-transmission models of environmental learning, prompting conceptual reorientation toward transformative, action-oriented, and competence-based pedagogies ( Kioupi & Voulvoulis, 2022; Sterling, 2021). Within this reorientation, science education has been reconceptualized as a privileged site for cultivating ecological reasoning, evidence-based decision-making, and civic agency directed at the resolution of socio-environmental problems ( Zeidler et al., 2019). The convergence of these imperatives signals a pressing scholarly need to examine which pedagogical configurations most effectively foster the dispositions and capacities demanded by sustainable futures, and to do so with the methodological rigor that the urgency of the problem warrants.

EL has emerged as an organizing construct capable of integrating ecological knowledge, cognitive and affective dispositions, behavioral competencies, and civic engagement into a coherent framework for sustainability-oriented learning ( Liang et al., 2022). Conceptually, it extends beyond informational ecological knowledge toward a relational understanding in which learners interpret the reciprocal couplings between human and natural systems, evaluate competing scientific and ethical claims, and translate that understanding into responsible environmental action ( Ardoin et al., 2020; Hadzigeorgiou & Skoumios, 2013). EL is closely affiliated with but analytically distinct from—sustainability literacy and ecological citizenship: whereas sustainability literacy foregrounds the integrative comprehension of economic, social, and environmental interdependencies, EL retains a distinctive focus on scientifically grounded engagement with biophysical systems and the ethical reasoning such engagement entails ( Cebrián et al., 2020; Reid, 2020). Recent scholarship has anchored EL to systems thinking, futures thinking, and normative competence as core sustainability competencies, arguing that fragmented disciplinary knowledge cannot adequately account for the wickedness and interdependence of contemporary ecological problems ( Brundiers et al., 2021; Redman & Wiek, 2021). This conceptual broadening has reinforced calls for pedagogies capable of moving beyond didactic transmission toward inquiry-driven, problem-saturated, and project-anchored learning environments in which EL can be enacted rather than merely transmitted.

PBL and PjBL, although rooted in distinct historical lineages—the former in clinical and medical education, the latter in Deweyan progressivism—have converged as paradigmatic student-centered pedagogies for sustainability and science education ( Markula & Aksela, 2022; Servant-Miklos, 2020). PBL situates learners within ill-structured, contextually authentic problems that demand iterative inquiry, hypothesis generation, and collaborative reasoning, whereas PjBL organizes learning around extended, driving-question-anchored projects that culminate in tangible artifacts or socio-environmental interventions ( Guo et al., 2020; Krajcik & Czerniak, 2021). Despite their pedagogical differences, both approaches share a constructivist commitment to authentic engagement, metacognitive reflection, and the redistribution of knowledge-generating agency from teacher to learner—features especially congenial to EL education, where the objects of study are by definition complex, contested, and value-laden. Empirical syntheses have credited PBL and PjBL with substantive gains in critical thinking, systems thinking, scientific argumentation, collaborative problem-solving, and pro-environmental dispositions ( Aksela & Haatainen, 2019; Almulla, 2020). More recently, both pedagogies have been positioned as institutional vehicles for embedding the SDGs and key sustainability competencies into curricular practice across educational levels ( Lozano et al., 2022; Membrillo-Hernández et al., 2021). Nevertheless, the conceptual articulation between PBL, PjBL, and EL remains under-theorized, frequently treated as an implicit byproduct of inquiry-based instruction rather than a deliberate design outcome anchored in the ESD agenda.

This conceptual under-articulation is especially acute in chemistry and environmental chemistry education, where PBL and PjBL hold considerable yet largely unrealized potential for cultivating molecular-level EL. Chemistry occupies a strategically central position within the science of sustainability: it explains the molecular mechanisms of climate change, the chemical bases of pollution and remediation, the principles of green chemistry, and the material flows that underpin both ecological degradation and ecological restoration. Yet the empirical literature on PBL- and PjBL-based EL education has gravitated overwhelmingly toward biology, environmental studies, and engineering, leaving chemistry education comparatively peripheral to the global ESD discourse. Mapping this asymmetry is a substantive contribution of the present study, since the consolidation of sustainability-oriented science education will require chemistry educators to engage more deliberately with active, problem-saturated, and project-anchored pedagogies ( Mahaffy et al., 2019). This design choice also justifies the use of a thematically focused, small-corpus bibliometric approach, consistent with precedent studies that have effectively mapped emergent subfields using comparable corpus sizes ( Passas, 2024).

A growing corpus of empirical research published between 2011 and 2026 has interrogated the educational efficacy of PBL and PjBL in environmental and sustainability contexts, yet the field remains methodologically and conceptually fragmented. Studies have reported gains across EL components—ecological knowledge, attitudes, behavioral intention, and competence—through interventions ranging from socio-scientific issue-based PBL in secondary science classrooms to community-anchored PjBL in higher education ( Genc et al., 2018; Suwono et al., 2023). Parallel work has explored the affordances of PjBL for STEM-integrated sustainability learning, place-based ecological inquiry, and SDG-oriented competency development ( Tomas et al., 2023). Across this literature, however, several gaps recur. First, PBL and PjBL are frequently conflated or used interchangeably in empirical reports, obscuring their differential affordances for cultivating distinct dimensions of EL ( Markula & Aksela, 2022). Second, despite the proliferation of intervention studies, comprehensive bibliometric mappings that trace the intellectual structure, collaborative networks, and thematic evolution of the triadic field linking PBL, PjBL, and EL are conspicuously absent ( Hallinger & Nguyen, 2020). Third, existing reviews tend to privilege either pedagogical efficacy or sustainability outcomes in isolation, rarely synthesizing methodological characteristics, educational levels, learning outcomes, and SDG alignments of empirical studies within an integrative analytical frame. Fourth, interdisciplinary and cross-national synthesis remains underdeveloped, constraining the transferability of findings across diverse curricular, cultural, and policy contexts.

Addressing these gaps requires an analytical strategy capable of simultaneously delineating the macro-structural contours of the field and examining the substantive characteristics of its empirical core. The present study therefore adopts an integrative bibliometric and systematic content analysis design—an approach increasingly recognized for advancing reflexive, evidence-informed scholarship in education and sustainability research ( Donthu et al., 2021; Kraus et al., 2022). Bibliometric mapping enables the quantitative reconstruction of publication trajectories, intellectual influence, and thematic clusters, while systematic content analysis affords a qualitative investigation of educational levels, learning approaches, learning outcomes, SDG integration, and methodological orientations within the empirical core of the field. Within this design, the study (i) analyzes the longitudinal publication trends of research on PBL, PjBL, and EL in educational contexts; (ii) identifies the most influential authors, institutions, and countries shaping the field; (iii) maps its dominant themes, keywords, and conceptual structures; (iv) examines patterns of international collaboration and co-authorship; (v) characterizes the empirical core through systematic content coding of educational level, learning approach, learning outcomes, SDG integration, and research methodology; and (vi) identifies emerging themes, research gaps, and prospective directions for future inquiry, with explicit attention to chemistry and environmental chemistry education as an under-developed sub-field. By foregrounding the convergence of PBL, PjBL, and EL within a single analytical frame, the study contributes a more coherent knowledge base for sustainability-oriented science education and offers researchers, educators, and policymakers a critical roadmap for advancing transformative environmental learning aligned with the 2030 Agenda.

## 2. Literature review

### 2.1. Environmental literacy in education

EL has evolved from a narrowly cognitive construct originally conceived as the public’s capacity to comprehend ecological information into a multidimensional educational outcome that integrates ecological knowledge, affective dispositions, cognitive and behavioral competencies, and civic engagement ( Ardoin et al., 2020). Contemporary scholarship characterizes the construct along at least four interlocking domains: substantive ecological knowledge of biophysical systems and human impacts; environmental attitudes and values that orient learners toward responsibility and stewardship; competencies for analyzing and acting on environmental problems, including systems thinking and critical reasoning; and pro-environmental behavior expressed through individual decisions and collective civic action ( Hadzigeorgiou & Skoumios, 2013; Liang et al., 2022). This expansion mirrors a broader epistemological shift in environmental and science education from information-transmission models toward action-competence frameworks in which knowing, valuing, and acting are conceptualized as mutually constitutive rather than sequentially ordered ( Rieckmann, 2018; Wals, 2020). Within ESD discourse, EL has accordingly been repositioned as a foundational but not exhaustive layer of sustainability-oriented capability, specifically attuned to scientifically grounded engagement with biophysical systems and the ethical reasoning such engagement entails.

Despite this conceptual maturation, the literature exhibits persistent inconsistencies that complicate empirical synthesis. EL is frequently conflated with adjacent constructs—sustainability literacy, ecological literacy, climate literacy, and environmental awareness—even though these constructs operate at different scales of integration: sustainability literacy foregrounds the systemic interplay of social, economic, and ecological dimensions, whereas EL retains a tighter analytical focus on biophysical systems and the science needed to interrogate them ( Cebrián et al., 2020; Reid, 2020). Measurement practices reflect this conceptual heterogeneity, with instruments differing markedly in dimensionality, item content, and the granularity at which behavioral and competence outcomes are operationalized (Genc et al., 2020; Liang et al., 2022). Such inconsistency hinders cross-study comparison, weakens claims of intervention efficacy, and obscures the conditions under which specific pedagogical approaches yield specific literacy gains. For the present study, this implies that any meaningful synthesis of PBL- and PjBL-based EL research must explicitly map how the construct is conceptualized, operationalized, and measured across studies, rather than treat it as a stable, uniformly defined outcome.

### 2.2. Problem-based learning and project-based learning

PBL and PjBL share a common epistemological lineage in social constructivism and situated cognition, but they were institutionalized through distinct historical trajectories. PBL was consolidated within clinical and medical education as a structured pedagogy for navigating ill-defined, contextually authentic problems through iterative inquiry, hypothesis generation, and collaborative reasoning, whereas PjBL evolved out of Deweyan progressivism and crystallized in K–12 science education around extended, driving-question-anchored projects that culminate in tangible artifacts or socio-environmental interventions ( Krajcik & Czerniak, 2021; Markula & Aksela, 2022; Servant-Miklos, 2020). Despite these differences, both pedagogies operate within a shared design logic in which learners function as knowledge-generating agents responsible for problematizing phenomena, mobilizing disciplinary knowledge, and producing publicly defensible claims or solutions. Recent empirical syntheses confirm that, when implemented with fidelity, PBL and PjBL enhance students’ critical thinking, scientific argumentation, collaborative problem-solving, self-regulated learning, and conceptual understanding across a range of disciplinary contexts ( Aksela & Haatainen, 2019; Almulla, 2020; Guo et al., 2020).

The pedagogical purchase of these approaches becomes especially salient in environmental and sustainability education, where learning objects are inherently complex, value-laden, and resistant to algorithmic resolution. By embedding learners in authentic socio-ecological problems, PBL and PjBL operationalize the dispositional, cognitive, and behavioral dimensions of EL that didactic instruction cannot easily reach, including systems thinking, anticipatory reasoning, normative judgment, and strategic action competence ( Brundiers et al., 2021; Lozano et al., 2022; Membrillo-Hernández et al., 2021). Within chemistry education in particular, PBL and PjBL hold latent potential for translating abstract molecular concepts into authentic environmental problem-solving, linking laboratory practice to real-world chemical issues such as water quality, atmospheric pollution, and waste valorization ( Mahaffy et al., 2019). At the same time, the empirical literature is marked by terminological slippage: studies routinely label structurally PjBL-like interventions as “PBL,” or conflate the two approaches under generic banners such as “inquiry-based” or “active learning,” obscuring the differential affordances each pedagogy holds for cultivating specific EL outcomes ( Markula & Aksela, 2022; Servant-Miklos, 2020). For the present study, this entanglement justifies treating PBL and PjBL as conceptually adjacent yet analytically separable in the bibliometric and content analyses, so that their distinct trajectories, communities, and outcome profiles within EL research can be made visible rather than collapsed.

### 2.3. ESD and the SDGs in education

ESD has matured from a peripheral curricular concern into a globally institutionalized educational paradigm, codified in the UNESCO ESD for 2030 framework and operationally aligned with SDG Target 4.7 ( Rieckmann, 2018). At its core, ESD is theorized as transformative rather than instrumental learning: it seeks to reorient cognitive structures, value commitments, and agentic capacities toward the resolution of complex sustainability problems, rather than merely augment students’ factual stock about environmental issues ( Wals, 2020). This transformative orientation has been operationalized through competence-based frameworks—most prominently the cluster of key sustainability competencies (systems, anticipatory, normative, strategic, interpersonal, intrapersonal, and integrated problem-solving competencies) consolidated by Brundiers et al. (2021) and the European GreenComp framework articulated by Bianchi et al. (2022)—which collectively reposition the learner as an agent of sustainability transitions. These frameworks share a pedagogical logic that privileges authentic, learner-centered, and inquiry-driven instruction, foregrounding precisely the design features that distinguish PBL and PjBL from didactic alternatives ( Cebrián et al., 2020; Redman & Wiek, 2021).

The translation of ESD and the SDGs into actual classroom and curricular practice, however, remains uneven and conceptually contested. Studies across higher education and school sectors document persistent gaps between rhetorical commitments to sustainability and the substantive integration of sustainability competencies into learning outcomes, assessment regimes, and instructional design ( Leal Filho et al., 2021; Lozano et al., 2022). Curricular fragmentation, weak teacher preparation in sustainability pedagogies, the disciplinary inertia of science curricula, and the difficulty of evaluating long-horizon, behavioral, and value-based outcomes consistently emerge as structural obstacles ( Corres et al., 2020; Tomas et al., 2023). Within this landscape, EL functions as a critical mediating construct: it provides the scientific and ethical scaffolding through which broader ESD competencies become tractable in classroom contexts, while inquiry-driven pedagogies such as PBL and PjBL provide the instructional architecture through which that literacy can be enacted. For the present study, this implies that the value of mapping PBL and PjBL research lies not only in pedagogical optimization but in clarifying the empirical pathways through which student-centered learning operationalizes the ESD agenda.

### 2.4. Previous bibliometric and review studies

A growing body of bibliometric and systematic review work has begun to chart the intellectual contours of sustainability- and environment-oriented education. Hallinger and Nguyen (2020) provided one of the most comprehensive bibliometric analyses of ESD, surfacing the field’s accelerating growth, geographic concentration in a small set of high-output countries, and thematic gravitation toward higher education and competence-based learning. Complementary syntheses have mapped educator competences for sustainability ( Corres et al., 2020), the outcomes of environmental education for conservation behavior ( Ardoin et al., 2020), and the implementation of sustainability education in secondary schooling ( Tomas et al., 2023). On the pedagogical side, systematic and meta-analytic reviews of PjBL in higher education have evidenced consistent positive effects on cognitive, affective, and behavioral outcomes, while signaling persistent methodological heterogeneity and weak attention to longitudinal impact ( Almulla, 2020; Guo et al., 2020). Methodologically, this expanding review literature has been catalyzed by maturing bibliometric guidelines ( Donthu et al., 2021; Kraus et al., 2022) and by the consolidation of analytic infrastructures such as Bibliometrix and VOSviewer that have democratized large-scale science mapping ( Aria & Cuccurullo, 2017).

Notwithstanding these advances, several intersecting gaps justify the present study. First, although bibliometric work exists on ESD, sustainability education, and—separately—on PBL or PjBL, no comprehensive analysis triangulates PBL, PjBL, and EL as a single intellectual field, leaving their shared knowledge structures, communities, and thematic trajectories largely invisible ( Hallinger & Nguyen, 2020). Second, existing reviews tend to privilege either pedagogical efficacy or sustainability outcomes, but rarely combine bibliometric mapping with systematic content analysis that interrogates educational levels, learning approaches, learning outcomes, SDG integration, and methodological orientations within an integrative frame ( Tomas et al., 2023). Third, interdisciplinary syntheses that connect science education, environmental education, and ESD scholarship around a shared pedagogical core remain underdeveloped, constraining the cumulative theory-building that sustainability-oriented science education requires ( Suwono et al., 2023; Zeidler et al., 2019). The present study addresses these gaps by integrating bibliometric science mapping with systematic content analysis, thereby providing both a macro-structural cartography of the PBL–PjBL–EL field and a substantive characterization of its empirical core—an integrative contribution that, to the best of our knowledge, has not been previously offered in the science education literature.

## 3. Methodology

### 3.1. Research design

This study adopted a sequential mixed analytical design that integrates bibliometric science mapping with systematic content analysis, an approach increasingly used in high-impact sustainability and education research for its capacity to combine the structural breadth of quantitative mapping with the substantive depth of qualitative interpretation ( Donthu et al., 2021; Linnenluecke et al., 2020; Mukherjee et al., 2022). Bibliometric analysis is here understood as a quantitative, citation- and metadata-driven examination of a scientific corpus that reconstructs publication trajectories, identifies influential actors and venues, surfaces patterns of intellectual influence and collaboration, and exposes the thematic architecture of a field through co-word, co-citation, and co-authorship networks ( Aria & Cuccurullo, 2017; Donthu et al., 2021). Science mapping, as a derivative analytical mode, complements descriptive bibliometrics by visualizing the relational geometry of knowledge production—rendering the conceptual, social, and intellectual structures of a domain in a form amenable to interpretation ( Cobo et al., 2011; Hallinger & Nguyen, 2020).

It should be noted that while the final corpus of 48 documents is modest in absolute terms, thematically focused bibliometric analyses operating with comparable or smaller corpora have demonstrated that meaningful structural and thematic patterns can be reliably identified when the corpus boundary is conceptually coherent rather than artificially inflated ( Passas, 2024). The present study’s corpus size reflects the genuine empirical boundaries of research explicitly connecting PBL/PjBL with environmental, ecological, or sustainability literacy, and the analytical outputs should accordingly be interpreted as descriptive cartographies of an emergent subfield rather than as statistically generalizable population parameters.

While bibliometric techniques are uniquely suited to delineating the macro-structural contours of a field, they are limited in their capacity to interrogate what individual studies actually do—how they frame learning outcomes, which educational levels they target, how they operationalize sustainability, and which methodological architectures they deploy ( Kraus et al., 2022; Snyder, 2019). Systematic content analysis was therefore introduced as a complementary analytical layer to address this interpretive deficit. By coding the empirical core of the corpus along theoretically grounded categories educational level, learning approach, learning outcomes, SDG integration, and research methodology the study produces an analytic surface upon which bibliometric findings can be substantively contextualized ( Krippendorff, 2018). The combined design is particularly appropriate for investigating the convergence of PBL, PjBL, and EL: a domain that is conceptually heterogeneous, methodologically diverse, and institutionally distributed across science education, environmental education, and ESD scholarship. Triangulating macro-level mapping with micro-level coding therefore enables a more reflexive and theoretically productive synthesis than either approach could yield in isolation.

### 3.2. Database selection

Scopus was selected as the sole bibliographic source on the basis of its breadth of indexed journals, the rigor of its source-selection criteria, the comprehensiveness of its citation metadata, and its established standing as the preferred database for bibliometric work in education, sustainability, and the social sciences ( Mongeon & Paul-Hus, 2016; Pranckutė, 2021). Relative to Web of Science, Scopus offers broader coverage of social science and education journals, which is essential for a study that intersects pedagogy, environmental education, and ESD scholarship; relative to Google Scholar, it offers the indexing discipline, deduplicated metadata, and citation traceability required for reproducible science mapping ( Donthu et al., 2021; Singh et al., 2021). Although dual-database designs combining Scopus and Web of Science are sometimes preferred for maximizing recall, single-source designs are widely accepted in the bibliometric literature when analytical consistency, metadata uniformity, and reproducibility are prioritized, and when the target field is well represented within the chosen database ( Echchakoui, 2020; Hallinger & Nguyen, 2020).

Three principled exclusions follow from this rationale. First, non-indexed databases and grey literature were excluded to preserve the peer-review threshold and to ensure that the analyzed corpus reflects the formally validated knowledge base of the field. Second, conference papers, editorials, errata, notes, and book chapters were excluded because their indexing depth, citation behavior, and editorial scrutiny differ systematically from peer-reviewed journal articles and reviews, introducing structural noise into citation- and co-word-based analyses ( Donthu et al., 2021; Pranckutė, 2021). Third, non-English publications were excluded to maintain analytical consistency in keyword extraction, abstract screening, and content coding, given the dominance of English in the indexed sustainability education literature; this exclusion is acknowledged as a limitation rather than treated as a neutral methodological choice. Together, these decisions privilege internal validity, metadata reliability, and analytical reproducibility over maximal recall—an appropriate trade-off for a thematically focused, conceptually integrative bibliometric study ( Linnenluecke et al., 2020; Passas, 2024).

### 3.3. Search strategy

The search strategy was designed to balance recall and conceptual precision through a Boolean intersection of two thematic blocks corresponding to the study’s analytical focus. The pedagogical block aggregated all common spelling and acronym variants of the target instructional approaches, while the conceptual block aggregated the principal literacy constructs through which environmental and sustainability competencies are theorized in the contemporary literature. The final query, executed in the TITLE-ABS-KEY fields of Scopus on 10 May 2026, was specified as follows: *(“problem based learning” OR PBL OR “problem-based learning” OR “project based learning” OR “project-based learning”) AND (“environmental literacy” OR “ecological literacy” OR “sustainability literacy”).*

The pedagogical string was deliberately constructed to capture both hyphenated and non-hyphenated orthographic conventions and to include the widely used acronym PBL, ensuring that variations in author practice and journal style would not produce systematic indexing exclusions ( Donthu et al., 2021; Linnenluecke et al., 2020). The conceptual string was constructed around three closely related but analytically distinct literacy constructs—environmental, ecological, and sustainability literacy reflecting the conceptual heterogeneity of the field documented in recent reviews ( Cebrián et al., 2020) and ensuring that thematic adjacency rather than terminological uniformity governed inclusion.

Search refinement proceeded through three sequential filtering stages. The initial execution of the pedagogical string within the publication window 2011–2026 yielded 34,822 documents. Restriction to English-language publications justified above on grounds of analytical consistency reduced the dataset to 33,133 documents. The Boolean intersection with the literacy-focused conceptual block, combined with restriction to article and review document types, produced a final, thematically focused dataset of 48 documents that constitute the analytical corpus of the study. The publication window 2011–2026 was deliberately chosen to encompass the period following the institutional consolidation of the United Nations Decade of ESD (2005–2014), the Global Action Programme on ESD (2015–2019), and the ESD for 2030 framework, thereby capturing the era in which sustainability-oriented pedagogies have been most actively theorized and empirically tested. The query date of 10 May 2026 ensured comprehensive coverage of all articles indexed through that date, including three studies bearing 2026 publication dates that had been indexed by Scopus prior to query execution. Sequential filtering, rather than a single all-encompassing query, was preferred because it permits transparent reporting of how each restriction affects yield, supports reproducibility, and minimizes the bibliometric noise that arises when conceptually heterogeneous documents are clustered together at scale ( Hallinger & Nguyen, 2020; Page et al., 2021a).

### 3.4. Inclusion and exclusion criteria

Inclusion and exclusion criteria were specified a priori and applied uniformly across the screening stages to ensure conceptual relevance, methodological consistency, and analytical rigor. Documents were retained only if they were peer-reviewed articles or reviews indexed in Scopus, written in English, published between 2011 and 2026, and substantively engaged with PBL or PjBL in connection with environmental, ecological, or sustainability literacy in an educational context (formal or non-formal, across primary, secondary, tertiary, or teacher-education settings). Studies were excluded if they (i) treated PBL or PjBL only incidentally without operationalizing the pedagogy as a core instructional design, (ii) addressed environmental or sustainability themes without a literacy-oriented learning construct, (iii) were situated outside educational contexts, or (iv) belonged to non-eligible document types. Duplicate records were screened automatically through Scopus export deduplication using EID matching and verified manually during the eligibility stage.

Beyond formal eligibility, thematic relevance was verified through structured title and abstract screening, followed by full-text inspection where ambiguity persisted. This dual-stage relevance screening reduces the risk of false positives that arise from keyword polysemy—particularly with respect to PBL, which can in some literatures denote production-based learning or play-based learning—and ensures that the analytical corpus reflects authentic engagement with the target pedagogical–conceptual nexus ( Kraus et al., 2022; Snyder, 2019). Table 1 summarizes the operational criteria.

**Table 1.** Inclusion and exclusion criteria.

Criterion	Inclusion	Exclusion
Database	Scopus-indexed records	Non-indexed sources, grey literature
Document type	Peer-reviewed article or review	Conference papers, book chapters, editorials, notes, errata, letters
Language	English	All other languages
Publication window	2011–2026	Records outside the window
Pedagogical focus	Substantive engagement with PBL or PjBL as the core instructional design	Incidental, rhetorical, or peripheral mention of PBL/PjBL
Conceptual focus	Environmental, ecological, or sustainability literacy as a learning outcome or guiding construct	Environmental/sustainability themes without a literacy-oriented construct
Context	Educational settings (formal or non-formal; any level)	Non-educational sustainability research, policy analyses without a learning component
Thematic relevance	Verified through title, abstract, and full-text inspection	Off-topic records identified through screening
Duplication	Unique records	Duplicate records identified via EID comparison

### 3.5. PRISMA flow process

The literature search and screening process adhered to the Preferred Reporting Items for Systematic Reviews and Meta-Analyses (PRISMA) 2020 statement ( Page et al., 2021a, 2021b), which constitutes the current de facto reporting standard for systematic and bibliometric reviews on account of its emphasis on procedural transparency, reproducibility, and methodological accountability. A completed PRISMA 2020 checklist and the corresponding PRISMA 2020 flow diagram are available as supplementary materials deposited in the Open Science Framework (OSF) repository (see Data Availability Statement; https://doi.org/10.17605/OSF.IO/XBDVA). Although PRISMA was originally developed for clinical evidence synthesis, its adaptation to bibliometric and educational reviews is now well established, providing a structured architecture within which identification, screening, eligibility, and inclusion stages can be reported with consistency ( Hallinger & Nguyen, 2020; Sianes et al., 2022; Tomas et al., 2023).

In the identification stage, an initial Scopus query using the pedagogical Boolean string within the 2011–2026 window returned 34,822 records, establishing the upstream pool from which subsequent filtering proceeded. In the screening stage, restriction to English-language publications reduced the dataset to 33,133 records, after which the Boolean intersection with the literacy-focused conceptual block, combined with restriction to article and review document types, narrowed the corpus considerably. EID-based deduplication confirmed that no within-database duplicates were present. In the eligibility stage, the remaining records were subjected to title and abstract screening against the inclusion and exclusion criteria detailed in Section 3.4, and ambiguous records were resolved through full-text retrieval and inspection. Records that operationalized PBL or PjBL only nominally, addressed sustainability outside an educational learning context, or used literacy in a non-environmental sense were removed at this stage. The final included corpus comprised 48 studies, which constituted the dataset for both the bibliometric science mapping and the systematic content analysis. The PRISMA 2020 flow diagram is presented in Figure 1.

**Figure 1.**
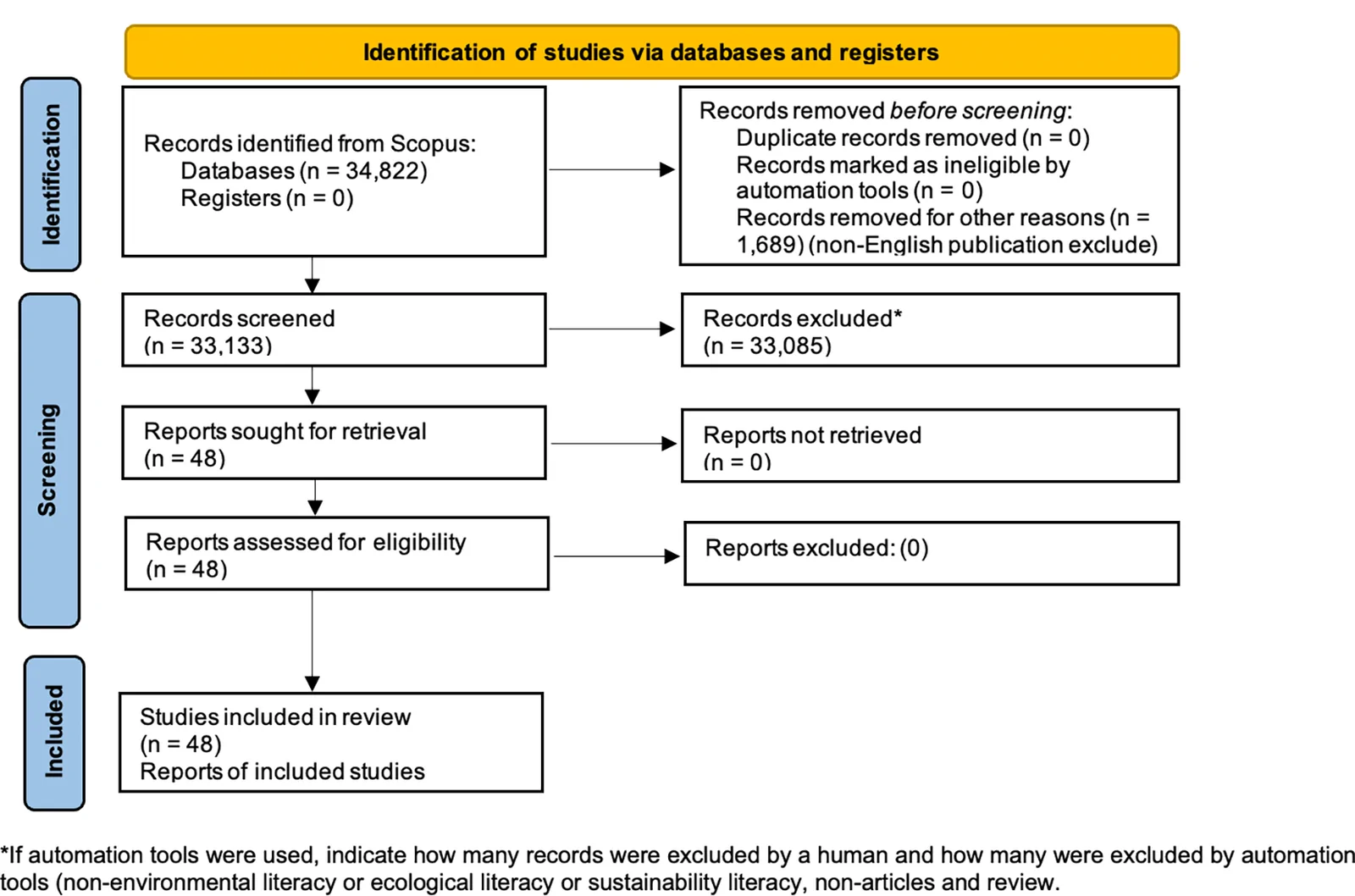
PRISMA 2020 flow diagram.

*If automation tools were used, indicate how many records were excluded by a human and how many were excluded by automation tools (non-environmental literacy or ecological literacy or sustainability literacy, non-articles and review.

Reporting these stages explicitly is more than a formal compliance exercise. PRISMA-anchored transparency is critical for bibliometric work because the analytical outputs—co-word maps, collaboration networks, thematic clusters—are highly sensitive to corpus boundaries; small differences in inclusion decisions can propagate into substantively different conclusions about the structure of a field ( Donthu et al., 2021; Page et al., 2021a). Documenting the journey from 34,822 records to 48 included studies thus supports replicability, allows other researchers to audit the analytical decisions, and clarifies the scope conditions under which the study’s findings should be interpreted.

### 3.6. Data analysis and analytical software

The analytical pipeline mobilized three complementary tools selected for their established methodological standing and their non-redundant analytical affordances: VOSviewer for bibliometric network visualization, Biblioshiny/Bibliometrix for science mapping and performance analysis, and Microsoft Excel for systematic content coding and frequency-based analysis. The combination follows the principle of analytical triangulation where each tool contributes a distinct epistemic angle, and convergent findings across tools strengthen the validity of inferences drawn from the corpus ( Donthu et al., 2021; Mukherjee et al., 2022).

VOSviewer ( van Eck & Waltman, 2010, 2017) was used to construct and visualize co-authorship and co-occurrence networks, applying its association-strength normalization and modularity-based clustering to identify communities of authors, institutions, countries, and keywords. Co-authorship analysis at author, institution, and country levels surfaced the social architecture of the field; keyword co-occurrence analysis surfaced its conceptual architecture; and overlay visualizations enabled the temporal interpretation of thematic shifts. The Biblioshiny web interface of the Bibliometrix R package ( Aria & Cuccurullo, 2017) was used in parallel to compute and visualize annual scientific production, source-level performance metrics, author productivity, country-level scientific production and collaboration intensity, three-fields plots linking authors, keywords, and countries, conceptual structure analysis through multiple correspondence analysis, and thematic mapping based on the centrality density plane ( Aria & Cuccurullo, 2017; Passas, 2024). Whereas VOSviewer excels at high-resolution network rendering, Bibliometrix excels at integrated performance and structural analytics within a unified statistical environment; using both therefore mitigates the analytical limitations of either tool in isolation ( Hallinger & Nguyen, 2020).

Microsoft Excel served as the operational environment for the systematic content analysis. A structured coding workbook was developed in which each of the 48 included studies was indexed against the analytical categories specified in Section 3.7. Excel was further used for frequency analysis of categorical variables (e.g., distribution of educational levels, learning approaches, and SDG alignments), cross-tabulation of categorical attributes, and the generation of summary descriptive statistics that complemented the network and performance analyses produced through VOSviewer and Bibliometrix. The deliberate combination of network analytics, performance analytics, and content-coded categorical analytics constitutes a layered methodological architecture in which each layer addresses a distinct analytical question what the structure of the field is, how it has evolved, and what its empirical core actually contains thereby producing a more reliable and theoretically productive synthesis than any single tool would permit ( Donthu et al., 2021; Linnenluecke et al., 2020).

### 3.7. Systematic content analysis

Systematic content analysis was applied to the 48 included studies to interrogate the substantive characteristics of the empirical core of the field beyond what bibliometric techniques can disclose. The analysis followed an integrated deductive–inductive coding logic in which an initial codebook was developed from the conceptual literature on EL, PBL, PjBL, and ESD, and was subsequently refined through iterative engagement with the corpus, allowing emergent categories to surface alongside theoretically anticipated ones ( Krippendorff, 2018). Five primary analytical categories structured the codebook: educational level (early childhood, primary, lower-secondary, upper-secondary, undergraduate, postgraduate, teacher education, non-formal); learning approach (PBL, PjBL, hybrid PBL/PjBL, blended with other inquiry-based pedagogies); learning outcomes (cognitive, affective, behavioral, and competence-based, with sub-categories drawn from established EL and sustainability competency frameworks); SDG integration (explicit alignment with one or more of the 17 SDGs, partial thematic alignment, or no explicit alignment); and research methodology (quantitative, qualitative, mixed-methods, with study-design sub-codes including quasi-experimental, case study, design-based research, action research, and survey).

## 4. Results and discussion

### 4.1. Publication trends

The longitudinal distribution of scholarly output on PBL, PjBL, and EL across 2011–2026 ( Figure 2) describes a field that remained latent for nearly a decade before undergoing a pronounced acceleration in the post-2021 period. Between 2011 and 2020, annual production fluctuated narrowly between zero and four articles, with two empty years (2013, 2014) and a transient spike in 2017—a pattern characteristic of an emergent rather than established research domain ( Hallinger & Nguyen, 2020). Three structural conditions plausibly account for this latency. First, PBL and PjBL had matured as pedagogies within institutionally distinct communities medical and engineering education for PBL, and K–12 science and STEM education for PjBL and these communities had limited routine engagement with EL scholarship, which itself remained anchored in environmental education and ecological psychology ( Markula & Aksela, 2022; Servant-Miklos, 2020). Second, the United Nations Decade of ESD (2005–2014) and the subsequent Global Action Programme (2015–2019) were oriented toward institutional advocacy and policy diffusion rather than empirical pedagogical experimentation, which delayed the translation of ESD discourse into classroom-level intervention research ( Wals, 2020). Third, EL was still being conceptually consolidated during this period, and the methodological infrastructure for measuring its multidimensional outcomes within active learning environments remained underdeveloped ( Ardoin et al., 2020). The transient 2017 peak likely reflects the educational reverberations of the 2015 Paris Agreement and the early operationalization of the SDGs in higher education curricula, but the absence of sustained growth thereafter signals that these stimuli had not yet produced a coherent research programme.

**Figure 2.**
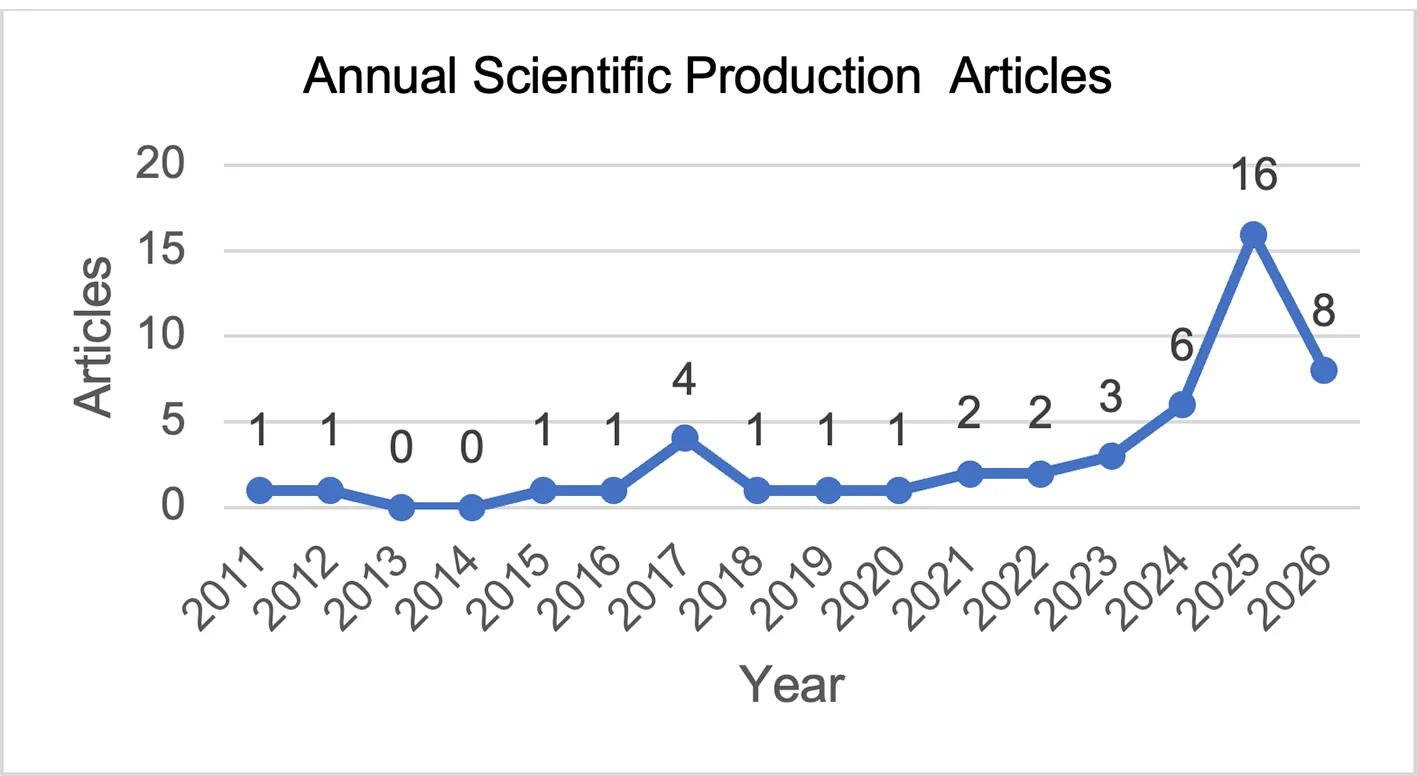
Annual scientific production on PBL/PjBL and environmental literacy.

The trajectory shifts decisively after 2020. Annual output doubles in 2021 (n = 2), holds in 2022, climbs to three articles in 2023, six in 2024, and reaches sixteen in 2025—a sixteen-fold increase over the 2020 baseline within five years. This acceleration is best interpreted as the empirical consequence of a series of converging institutional and intellectual movements rather than as a stochastic uptick. The launch of the ESD for 2030 framework, formalized through the 2021 Berlin Declaration, repositioned learning as a central lever of sustainability transformation and explicitly called for participatory, action-oriented, and competence-based pedagogies ( Rieckmann, 2018). The consolidation of sustainability competency frameworks—most prominently the reference framework articulated by Brundiers et al. (2021) and the European GreenComp framework ( Bianchi et al., 2022)—provided the conceptual scaffolding necessary to operationalize EL within structured pedagogical designs, and PBL and PjBL emerged as natural instructional vehicles for this operationalization ( Cebrián et al., 2020; Lozano et al., 2022). At the same time, the COVID-19 disruption catalyzed a broader scholarly reflection on the systemic vulnerabilities of contemporary societies and on the role of transformative education in cultivating ecological resilience, intensifying institutional and disciplinary pressure to integrate sustainability into curriculum design ( Kioupi & Voulvoulis, 2022; Leal Filho et al., 2021). The post-2021 surge therefore represents a phase transition in which the latent conceptual convergence between active pedagogies and EL was finally institutionally enacted in empirical research.

Figure 3, modeled as a Gaussian life-cycle curve fitted to the annual production series (R ^2^ = 0.78), localizes the modeled peak at 2025.2. The coefficient of determination indicates that approximately 78% of the variance in annual output is captured by the life-cycle specification—a robust fit by social science standards, particularly for a corpus of this magnitude—yet it is essential to interpret the modeled peak with interpretive caution. First, life-cycle modeling on small corpora (n = 48) tends to over-fit transient acceleration as terminal maturation, projecting a downward limb that is theoretically unwarranted in a field still consolidating its institutional infrastructure ( Aria & Cuccurullo, 2017; Hallinger & Nguyen, 2020). Second, even with the 2025 cut-off in place, the modeled peak should be read as a descriptive inflection rather than a predictive maturation point. Read against these caveats, the 2025.2 peak should not be interpreted as imminent saturation but rather as a mid-range inflection at which the field transitions from emergence into a sustained expansion-and-consolidation phase. The high goodness-of-fit alongside the substantively conditioned modeling assumptions thus jointly indicate a field that is rapidly maturing, gaining thematic coherence, and accumulating critical mass for theory-building—conditions consistent with the empirical signatures of newly institutionalized research domains in sustainability education ( Sianes et al., 2022).

**Figure 3.**
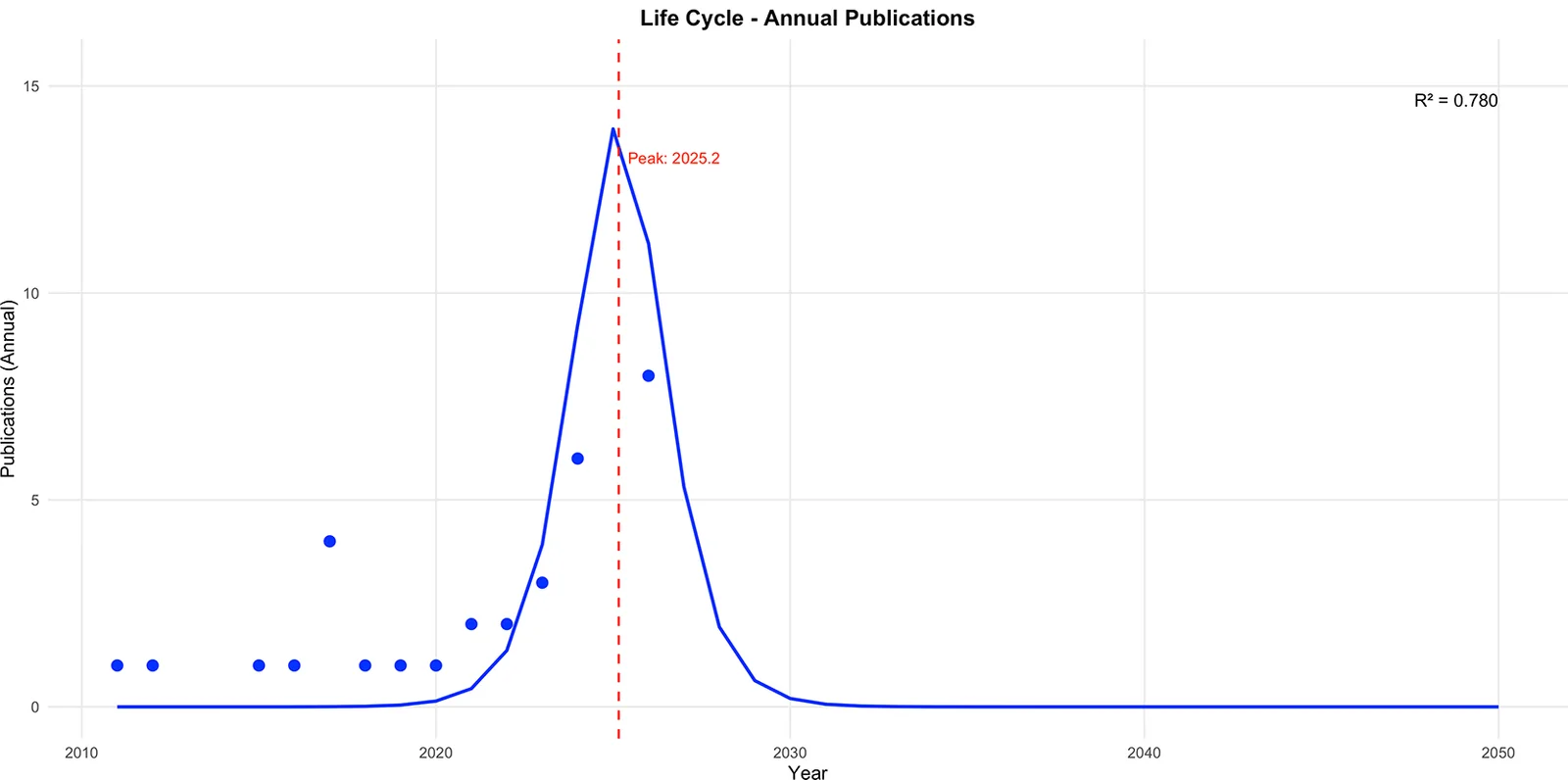
Life-cycle modeling of annual publications (Gaussian fit, R ^2^ = 0.78).

Figure 4 reinforces this interpretation by rendering the cumulative trajectory of publications as a logistic-style growth curve characterized by an extended low-output base, a steep inflection between 2023 and 2025, and a near-asymptotic approach to the corpus ceiling thereafter. The 50% cumulative threshold is crossed in 2024 and the 90% threshold in 2025, indicating that the majority of all scholarly output on PBL, PjBL, and EL has been produced within the most recent two-year window. Such compressed cumulative growth is diagnostic of intensified scholarly attention and rapid thematic consolidation, but it also signals the increasing interdisciplinarity of the field, in which contributions originate from science education, environmental education, ESD, sustainability science, and teacher education rather than from a single disciplinary base ( Hallinger & Nguyen, 2020; Tomas et al., 2023). The mainstreaming of student-centered, inquiry-driven, and project-anchored pedagogies into sustainability education appears to be the principal mechanism driving this acceleration, as PBL and PjBL provide the instructional architecture through which the multidimensional construct of EL encompassing ecological knowledge, systems thinking, normative competence, and pro-environmental action—can be enacted rather than merely transmitted ( Brundiers et al., 2021; Markula & Aksela, 2022; Wals, 2020).

**Figure 4.**
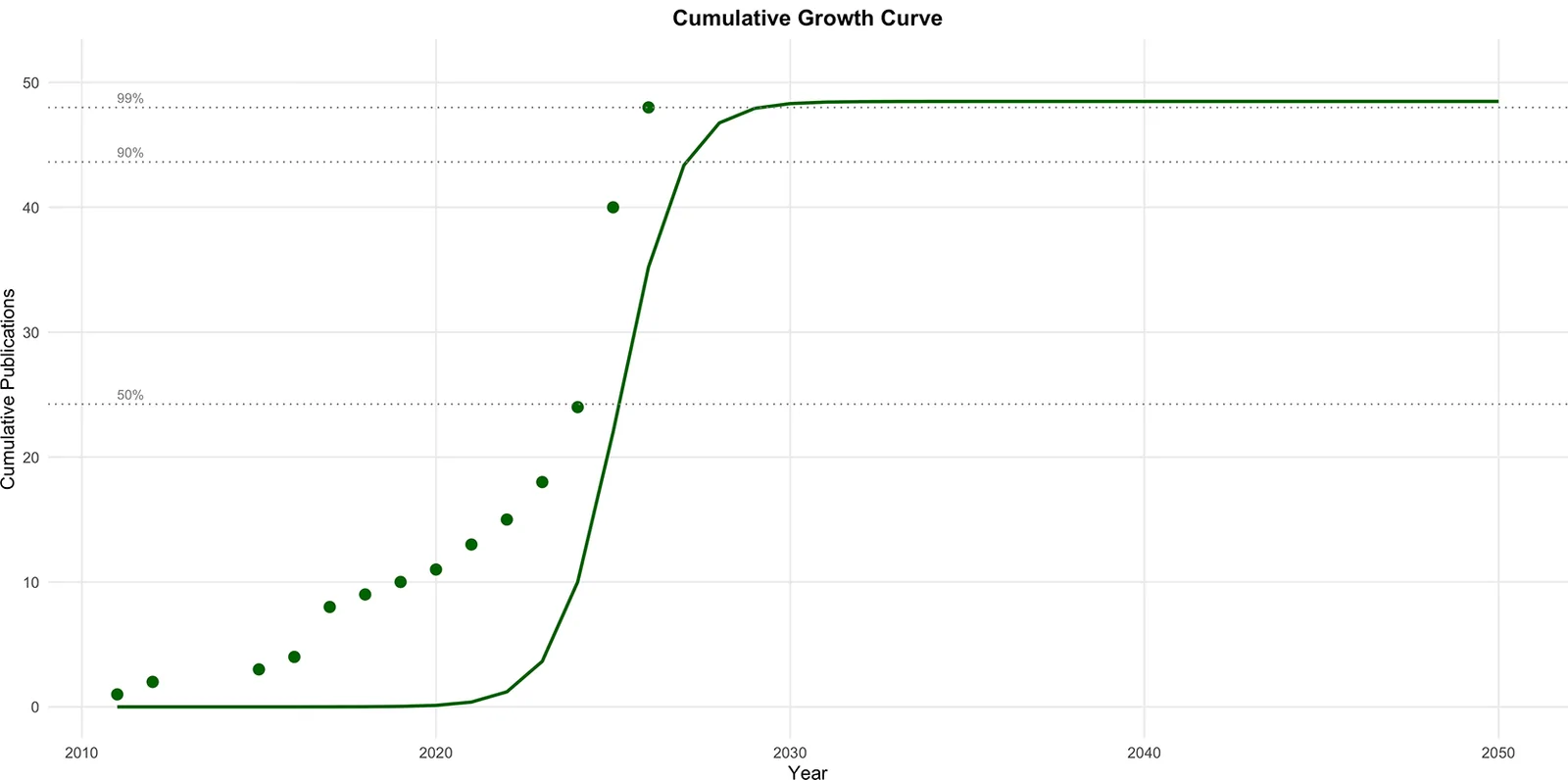
Cumulative growth curve of publications.

Taken together, Figures 2– 4 describe a research field that has moved within a single decade from intellectual latency to institutional visibility, mirroring the broader transformation of science and sustainability education from disciplinary segmentation toward integrated, competence-oriented, and transformative learning paradigms ( Rieckmann, 2018; Sterling, 2021; Wals, 2020). Three implications follow. First, the temporal coupling between the post-2021 acceleration and the institutional momentum of the ESD for 2030 framework, the GreenComp framework, and the SDG mid-term review suggests that the field is increasingly governed by policy-driven research agendas, with both the productive potential and the conformity risks that such governance entails. Second, despite the rapid quantitative expansion, conceptual and methodological gaps persist: the conflation of PBL and PjBL in empirical reports, the under-representation of long-horizon and design-based methodologies, and the limited articulation between specific active-pedagogy configurations and specific EL outcomes remain unresolved ( Markula & Aksela, 2022; Servant-Miklos, 2020). Third, the cumulative concentration of output within 2024–2025 indicates that the field is theoretically young, and its longer-term trajectory will depend on whether the current expansion produces cumulative theoretical synthesis or fragments into a constellation of context-specific intervention studies. The publication-trend evidence therefore both validates the timeliness of the present bibliometric and systematic content analysis and establishes the analytical imperative for the deeper structural, thematic, and substantive interrogations developed in the subsections that follow.

### 4.2. Most influential institutions and countries

The temporal mapping of affiliation productivity presented in Figure 5 discloses a markedly stratified institutional landscape, in which a small number of universities have come to anchor the global scholarship situated at the intersection of PBL, PjBL, and EL. While the University of California maintains a relatively flat but consistently high output throughout the period—reflecting the cumulative weight of an established, well-resourced research tradition—the most striking signal in the graph is the post-2017 acceleration of Universitas Negeri Malang, which converges with the leading institutions by 2025 at approximately six publications. Kennesaw State University exhibits a more stable productivity profile, suggesting a mature research line rather than a recent surge, whereas Universitas Pendidikan Indonesia, Universitas Negeri Padang, Sebelas Maret University, The University of Texas at Tyler, and Chirchik State Pedagogical University display more recent emergence. Read against the broader literature on higher education sustainability, this pattern indicates that institutional influence in this field is no longer determined solely by historical prestige; rather, it is being reshaped by universities that strategically invest in student-centered, inquiry-driven pedagogies aligned with the SDGs and with national environmental education priorities. In this sense, dominant institutions function less as repositories of disciplinary tradition than as agents of sustainability-oriented curriculum transformation, reaffirming the role of higher education as a structural lever for advancing ESD ( Leal Filho et al., 2021; Sterling, 2021).

**Figure 5.**
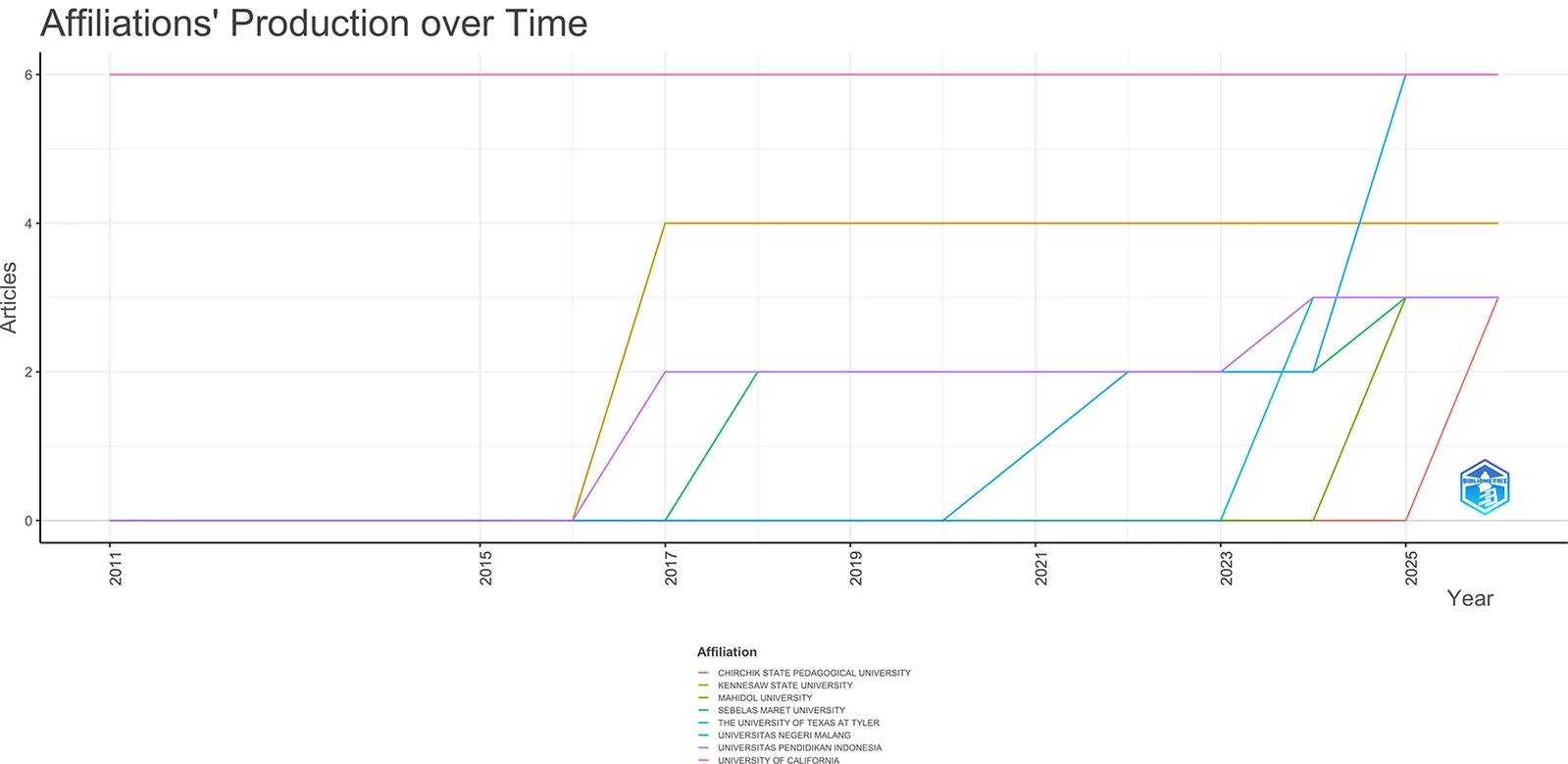
Affiliations’ production over time.

Figure 6 sharpens this picture by revealing a substantive geopolitical reconfiguration of the field. Indonesia has undergone the most dramatic publication acceleration, particularly after 2023, surpassing long-established contributors such as the United States and the United Kingdom, both of which display comparatively stable but unspectacular trajectories. Smaller yet rising contributions from Brazil, Germany, Thailand, Mexico, and Malaysia indicate a gradual broadening of the geographic base. Indonesia’s ascent cannot be adequately explained by demographic scale alone; rather, it reflects a confluence of policy instruments and academic incentives, including the integration of ESD into the Kurikulum Merdeka, the institutional reach of the Adiwiyata environmental schools program, and the alignment of national grant priorities with SDG 4 (Quality Education) and SDG 13 (Climate Action). This dynamic is consistent with broader evidence that UNESCO’s roadmap for ESD 2030 has substantially energized scholarly production in middle-income countries ( Wals, 2020). The asymmetry between developed and developing nations thus inverts the familiar assumption that sustainability research is primarily a Global North enterprise. Instead, it suggests that the locus of pedagogical innovation in EL is migrating toward research economies whose societies are simultaneously confronting acute ecological vulnerabilities, rapid demographic pressures on natural systems, and ambitious national agendas for educational transformation.

**Figure 6.**
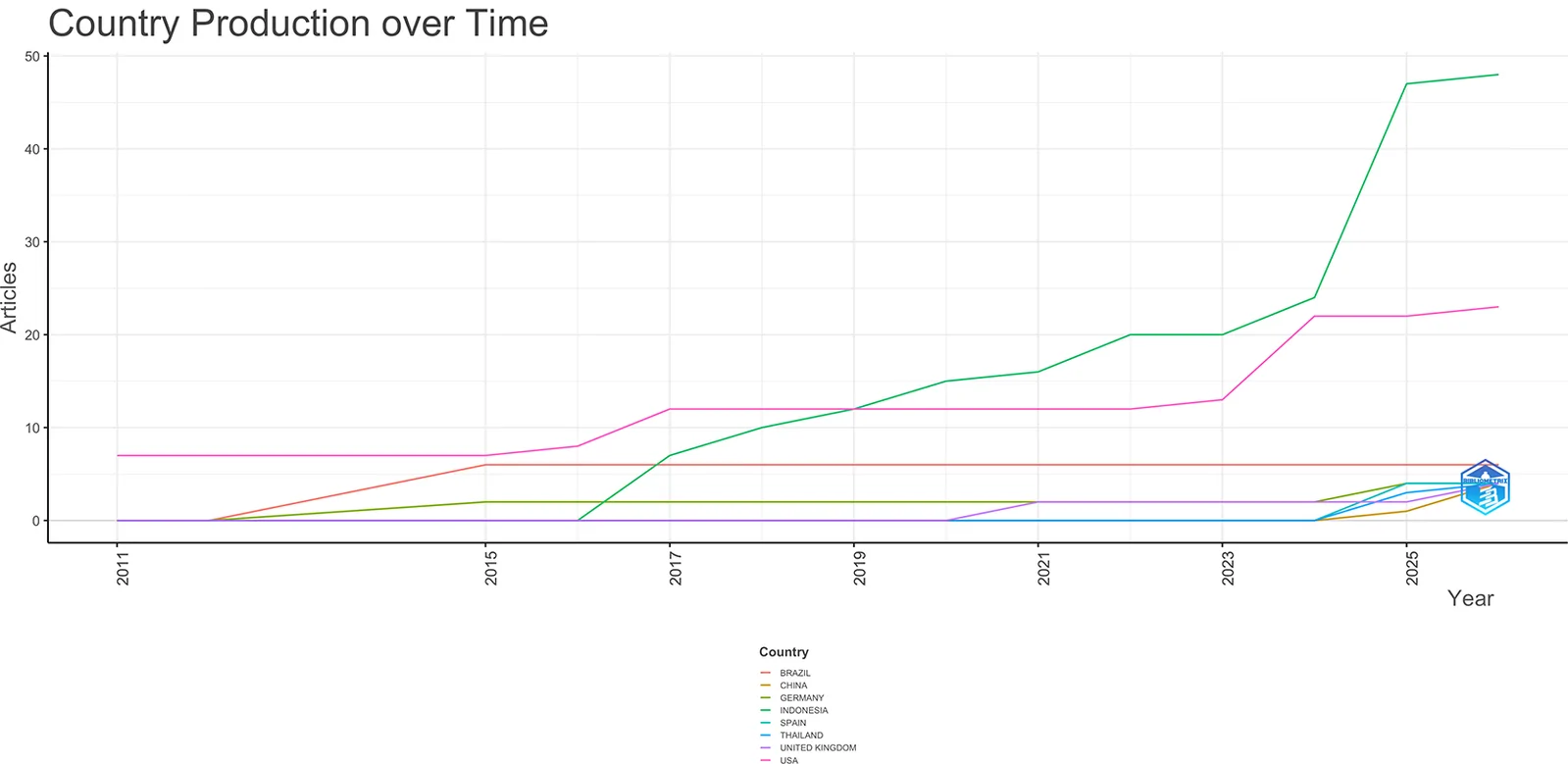
Country production over time.

The relational architecture depicted in Figure 7 deepens this interpretation by exposing how scholarly cooperation is being structured across the field. The Sankey configuration reveals dense flows between Indonesian authors and Indonesian institutions—particularly Universitas Negeri Malang, Universitas Pendidikan Indonesia, Universitas Negeri Jakarta, and Universitas Negeri Padang—alongside substantial linkages mediated by the United States through institutions such as the University of California, Kennesaw State University, and The University of Texas at Tyler. Brazil, anchored by Universidade Federal do Rio Grande do Sul, occupies a meaningful but secondary position, while Germany (Technische Universität München), Thailand, Uzbekistan, Mexico, and Malaysia appear as peripheral but active nodes. Taken together, the network is neither fully centralized nor genuinely globally distributed; rather, it constitutes a bipolar configuration in which two principal anchors—an established Northern hub and an ascendant Southern hub—coordinate a widening cluster of partners. Such structures are characteristic of what bibliometric scholars describe as transitional collaboration networks, in which knowledge co-production is mediated through asymmetric capacity-building partnerships and emerging regional clusters ( Donthu et al., 2021; Hallinger & Nguyen, 2020). The relative thinness of intra-Asian, intra-African, and South–South ties further indicates that interdisciplinary sustainability education research has not yet matured into a multipolar ecosystem.

**Figure 7.**
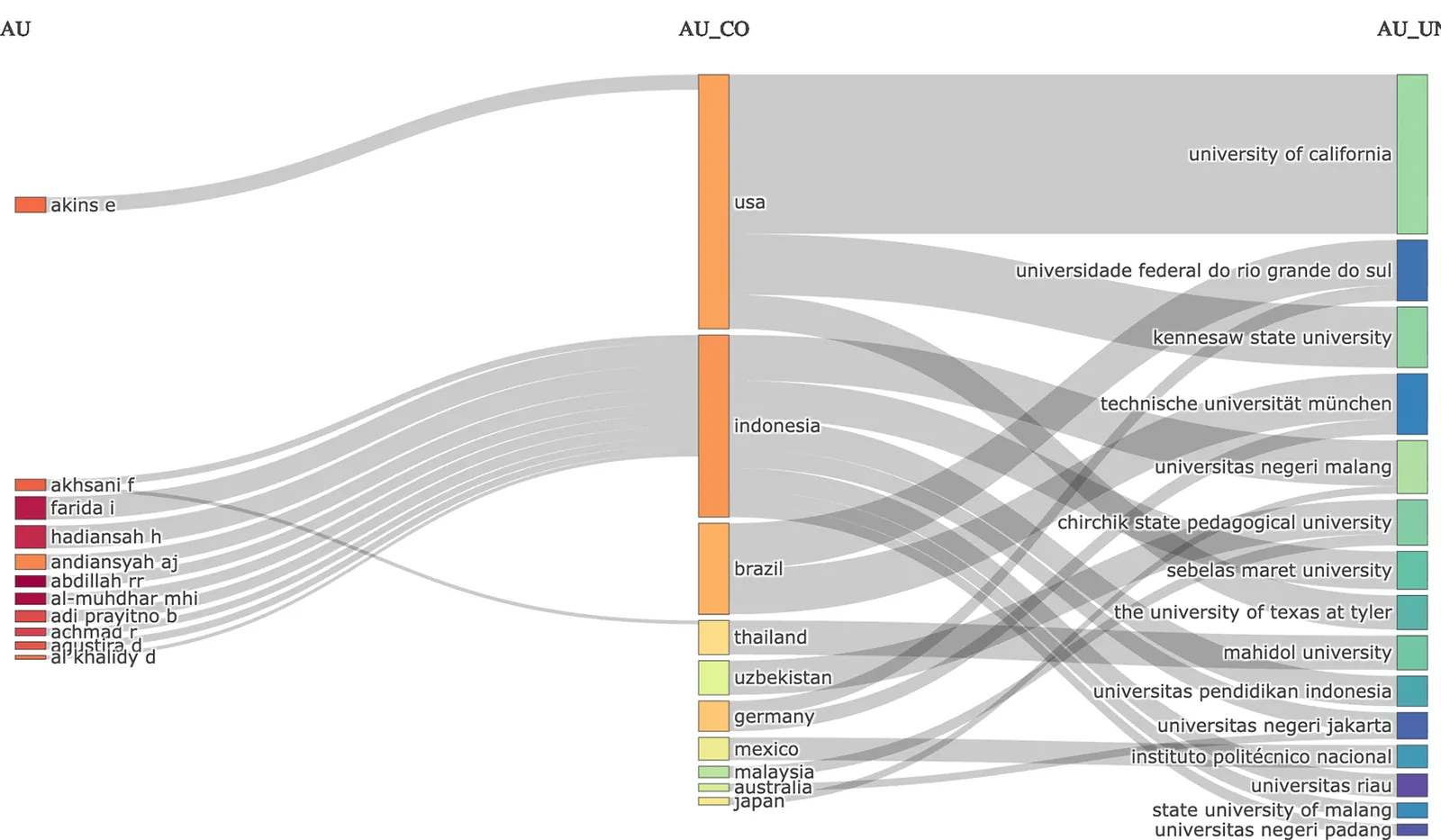
Three-fields plot of authors, countries, and institutions.

Beyond their descriptive value, these institutional and country-level patterns invite a more searching geopolitical and epistemological reading. The continuing concentration of high-impact venues, indexing infrastructures, and editorial gatekeeping within Global North academia generates structural barriers that constrain how EL scholarship from the Global South enters the canonical record ( Demeter, 2020; Mêgnigbêto, 2013). Linguistic hegemony in English-medium publishing, uneven access to indexed journals, disparities in competitive research funding, and limited embedding within established citation networks collectively shape what counts as legitimate sustainability knowledge. The Indonesian surge, while substantively important, may be interpreted as a partial countervailing force against this asymmetry, made possible by national investments in scientific publishing infrastructure, Sinta–Scopus alignment policies, and performance-based academic incentives. Yet this progress remains uneven across the Global South, as many countries facing the most pressing socio-ecological challenges remain underrepresented in the bibliometric record. Framed through the lens of environmental justice, this configuration raises ethical concerns: communities most exposed to ecological precarity continue to exercise limited authorship of the pedagogical knowledge intended to address those very vulnerabilities, thereby weakening the inclusivity that should anchor a just sustainability transition ( Kopnina, 2020; Leal Filho et al., 2021).

These institutional and geopolitical patterns both converge with and diverge from comparable bibliometric analyses. Hallinger and Nguyen (2020), mapping ESD research broadly, identified a field dominated by European and North American institutions with limited Global South participation; the present findings confirm that North American institutions retain structural importance but reveal a pronounced Indonesian ascendancy not captured in that earlier analysis, likely because the PBL/PjBL–EL nexus is a more recent phenomenon. Similarly, Fauzi and Wijarini (2024), in a bibliometric review of PBL and EL in science education, reported a comparable post-2020 publication surge and identified Indonesia as an emerging contributor, but their analysis did not extend to thematic evolution mapping or systematic content coding of the corpus. The present study extends both prior analyses by demonstrating that the field’s institutional geography is not merely expanding but structurally reconfiguring, with Indonesian universities transitioning from peripheral participants to major anchoring nodes within the global collaboration network. Tomas et al. (2023), reviewing sustainability education in secondary schools, noted a persistent under-representation of primary education and a dominance of higher education contexts, a pattern that the present content analysis corroborates across the PBL/PjBL–EL subfield specifically.

Synthesizing these findings, the institutional and country-level dynamics observed indicate that EL research grounded in PBL and PjBL is undergoing a phase of structural transformation, characterized by the simultaneous consolidation of established Northern centers and the accelerating rise of Global South contributors. This dual movement carries significant implications for the future trajectory of sustainability-oriented science education research. On one hand, the growing interdisciplinarity of the field—linking environmental science, pedagogy, curriculum studies, teacher education, and policy analysis—creates fertile ground for transformative learning frameworks aligned with the SDGs. On the other hand, the durability and equity of this growth will depend on whether emerging hubs in Indonesia, Latin America, and Southeast Asia can move beyond bilateral dependencies and cultivate horizontal South–South and South–North–South partnerships capable of generating knowledge that is simultaneously contextually grounded and globally legible. Universities, as the principal sites where pedagogical innovation, sustainability scholarship, and SDG localization converge, are therefore strategically positioned in this evolution. Strengthening collaborative infrastructures, supporting open-access dissemination, and embedding EL within institutional sustainability agendas are not auxiliary tasks but central conditions for advancing transformative environmental learning at a planetary scale ( Wals, 2020).

### 4.3. Keyword Co-occurrence analysis

Figure 8 presents the keyword co-occurrence network, comprising four distinct thematic clusters whose density and cross-cluster linkages jointly reveal the intellectual architecture of research on PBL, PjBL, and EL education. The overall topology is characterized by a dense, highly interconnected core—anchored by the high-frequency nodes “education,” “students,” “sustainability,” “problem-based learning,” and “project-based learning”—surrounded by more peripheral but theoretically significant terms. This structural pattern is consistent with a maturing, interdisciplinary field in which a stable conceptual core has formed, yet thematic differentiation is still actively occurring across methodological, contextual, and sustainability-theoretical dimensions ( Donthu et al., 2021; Zupic & Čater, 2015). The cross-cluster connections visible in the figure further indicate that no single thematic trajectory operates in isolation; rather, research on EL has evolved as an integrative enterprise linking pedagogy, curriculum, ecological theory, and sustainability competency development.

**Figure 8.**
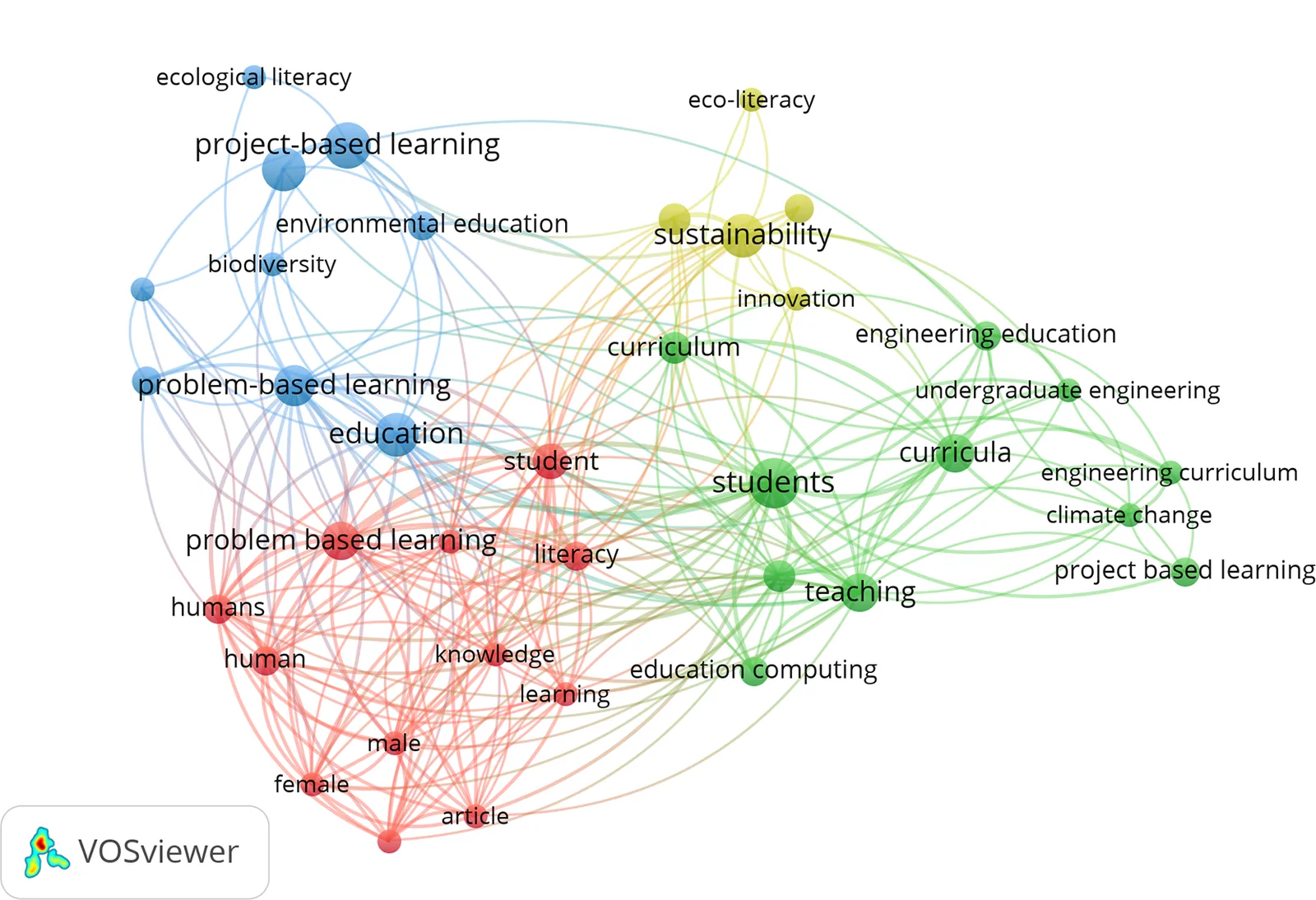
Keyword co-occurrence network.

The blue cluster—encompassing project-based learning, problem-based learning, environmental education, biodiversity, and ecological literacy—represents the foundational epistemological core of the field: experiential, inquiry-based environmental pedagogy grounded in constructivist and ESD principles. The co-occurrence of PBL and PjBL with ecological literacy and biodiversity signals that authentic environmental problem-solving, rather than content transmission, has become the dominant pedagogical logic for EL development ( Hmelo-Silver, 2004; Krajcik & Shin, 2014). This alignment is theoretically coherent: both PBL and PjBL place students in the role of active agents confronting ill-structured, real-world ecological problems—an instructional design that operationalizes Dewey’s (1986) principle of learning through experience and supports what Sterling & Orr (2001) describes as transformative sustainability education. The presence of biodiversity and ecological literacy as satellite terms reinforces the cluster’s orientation toward systems-level ecological understanding, moving beyond surface environmental awareness toward the deeper competency Lewinsohn et al. (2015) identify as essential for future environmental professionals. The blue cluster’s central position in the network confirms that PBL/PjBL-driven environmental pedagogy constitutes the intellectual nucleus from which the field’s other thematic concerns radiate.

The green cluster—comprising students, teaching, curricula, engineering education, undergraduate engineering, engineering curriculum, climate change, and project-based learning—reflects the field’s significant expansion into higher education and professional sustainability training. The co-occurrence of engineering-specific terms with curricula and climate change signals a curricular transformation agenda: universities, particularly engineering faculties, are increasingly repositioning their programs around SDG-aligned sustainability competencies rather than purely technical knowledge ( Leal Filho et al., 2021; Wiek et al., 2011). This trend is consistent with the influence of accreditation frameworks such as ABET’s sustainability criteria and the Engineering for One Planet initiative, which mandate measurable sustainability outcomes in undergraduate curricula ( Kwaczala et al., 2024). The prominence of climate change within this cluster is analytically significant: it confirms that climate education has migrated from peripheral elective content toward core curricular territory in higher education, driven by SDG 13 imperatives and growing institutional accountability for sustainability outcomes ( Barth et al., 2022). The green cluster thus maps the institutionalization of sustainability-oriented pedagogy at the level of curriculum design and professional education reform. Notably, however, chemistry-specific or environmental-chemistry-specific keywords are conspicuously absent from this cluster—an absence that mirrors and corroborates the under-representation of chemistry education in the broader corpus and signals a strategic gap for future inquiry.

The red cluster—containing student, literacy, knowledge, learning, human, humans, male, female, and article—captures the empirical, assessment-oriented dimension of the field, indexing studies primarily concerned with measuring learning outcomes and operationalizing EL as a cognitive or behavioral construct. The co-occurrence of literacy and knowledge with demographic descriptors (male, female) and methodological markers (article, humans) suggests that quantitative, survey-based, quasi-experimental designs dominate this research strand—studies that measure pre-post gains in environmental knowledge or literacy scores across stratified student samples ( Hollweg et al., 2011). While this cluster represents the field’s most empirically productive strand, it also reveals its most significant conceptual limitation: by operationalizing EL primarily as measurable knowledge acquisition, it risks reducing a complex, transformative construct to a psychometric variable, obscuring the behavioral, civic, and systemic dimensions that ESD frameworks foreground ( Roth, 1992; Rieckmann, 2017). The yellow cluster—containing sustainability, curriculum, innovation, and eco-literacy—occupies a bridging position between the blue and green clusters, conceptually representing the field’s sustainability-theoretical aspirations. The co-occurrence of eco-literacy, innovation, and curriculum signals an emerging research agenda concerned with designing educational experiences that cultivate holistic sustainability literacy rather than discrete environmental knowledge—an agenda aligned with Wals’ (2010) vision of social learning for sustainability and with the Rieckmann (2017) sustainability competency framework’s emphasis on anticipatory, normative, and systems-thinking competencies.

Taken together, the four-cluster structure of Figure 8 maps a field in productive but uneven development. The cross-cluster bridges visible in the network—particularly the links between the blue PBL/PjBL core and both the green curriculum-reform cluster and the yellow sustainability-innovation cluster—indicate that active learning approaches are increasingly being theorized within broader institutional transformation and sustainability competency frameworks rather than as isolated instructional methods ( Barth et al., 2022; Rieckmann, 2018). Nevertheless, the network reveals several intellectual gaps warranting critical attention. The absence of explicit SDG-labeled nodes and terms such as systems thinking, transformative learning, environmental citizenship, or sustainability competencies from the high-frequency core indicates that, while sustainability themes permeate the literature thematically, the field has not yet converged on a coherent theoretical vocabulary connecting PBL/PjBL to the broader ESD competency agenda ( Jickling & Wals, 2008; Vare & Scott, 2007). The dominance of the red cluster’s assessment-oriented keywords further signals an imbalance between empirical implementation research and theoretical sustainability framework development—a gap that limits the field’s capacity to build cumulative, transferable knowledge about how active pedagogies contribute to long-term sustainability transformation. Future research should prioritize conceptual integration across these clusters, explicitly situating PBL and PjBL within SDG-aligned competency frameworks and developing longitudinal, mixed-method research designs capable of capturing the full depth and duration of sustainability-oriented EL development.

### 4.4. Co-authorship analysis

Figure 9 presents the author collaboration network derived from co-authorship analysis, revealing a moderately fragmented yet selectively interconnected scholarly structure. Rather than forming a single dense community, the network consists of multiple discrete collaborative clusters—each anchored by a high-centrality author—linked by sparse but strategically important cross-cluster ties. The most prominent nodes include Abdillah RR, Al-Muhdhar MHI, Flight C, Engels J, Bruno BC, Cackowski C, Böttjer-Wilson D, and Bennett A, whose comparatively larger node sizes and multi-colored connecting lines indicate elevated co-authorship frequency and thematic reach across different research groups. Abdillah RR and Al-Muhdhar MHI form a tightly bonded dyad embedded within a broader Indonesian cluster alongside Farida I, Hadiansah H, Agustira D, and Akhsani F—a configuration consistent with institution-level research groups at Universitas Negeri Malang that concentrate on PBL-driven environmental and ecological literacy development. On the opposing side of the network, Bruno BC, Cackowski C, Bennett A, and Böttjer-Wilson D constitute a North American cluster whose collaborative ties reflect interdisciplinary applied ocean and environmental science education projects. The cross-cluster linkages—represented by the long arching lines connecting Indonesian and North American nodes via intermediate authors such as Engels J and Flight C—are analytically significant: they suggest that, while regional research communities operate with substantial internal cohesion, a thin but functional layer of transnational collaboration exists, facilitating methodological and thematic exchange between the Global South and the Global North ( Donthu et al., 2021). Overall, however, the network’s predominantly fragmented topology indicates that the field has not yet achieved the integrative, multi-hub collaboration structure characteristic of mature interdisciplinary research domains ( Zupic & Čater, 2015), representing a structural constraint on cumulative knowledge-building in sustainability-oriented EL education.

**Figure 9.**
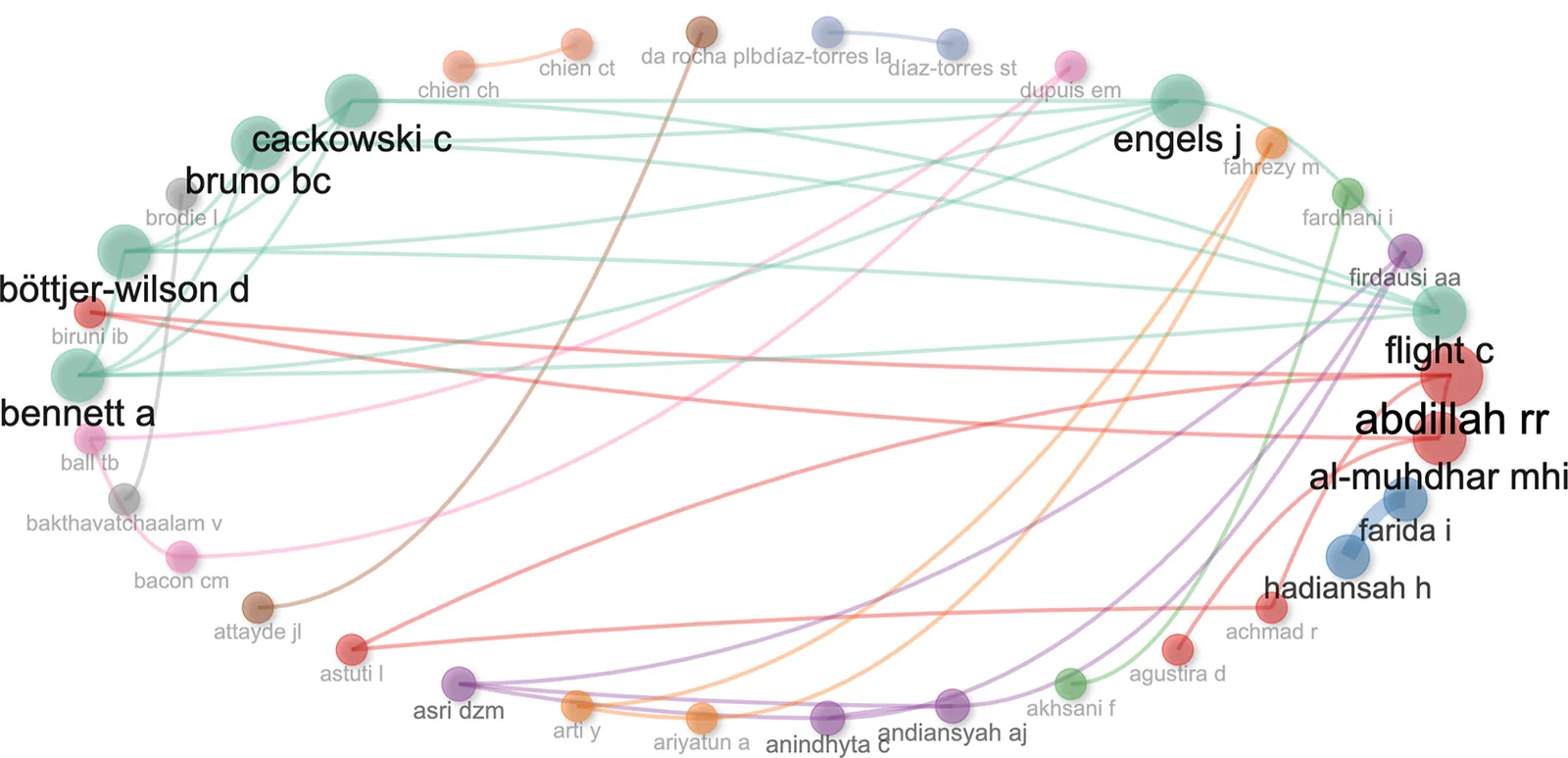
Author collaboration network.

Figure 10 complements this structural reading with a geopolitical dimension. Indonesia is unambiguously the dominant corresponding-author country, contributing approximately 17 documents—the large majority classified as Single Country Publications (SCP), with a smaller but non-negligible Multiple Country Publications (MCP) component. The United States ranks second with approximately five documents, nearly all SCP, followed by China and Thailand, both of which exhibit notably higher MCP ratios relative to their total output, suggesting stronger international co-authorship orientation despite lower absolute productivity. Canada, Germany, Mexico, Moldova, Slovenia, Spain, the United Kingdom, and Uzbekistan each contribute one to two documents, mostly SCP. The SCP-to-MCP ratio is diagnostically important: a high SCP share, as observed for Indonesia, reflects robust national research capacity and publication activity within sustainability and EL education, yet simultaneously signals limited integration into global co-authorship networks, a pattern consistent with the productivity–isolation paradox identified in Global South bibliometric analyses. The United States’ exclusively SCP profile is equally noteworthy: despite its strong global research visibility, American researchers in this corpus appear to operate largely within domestic collaborative frames rather than driving international sustainability education partnerships. Conversely, China’s and Thailand’s higher MCP proportions suggest that researchers from these countries actively leverage cross-border collaboration to extend their international scholarly reach, a strategy increasingly common among Asian institutions seeking Scopus-indexed visibility ( Leal Filho et al., 2021). The under-representation of African, Latin American (beyond Mexico), and South Asian countries—regions acutely affected by environmental degradation and where EL deficits are most consequential—constitutes a significant equity gap in the global architecture of sustainability education research, one that reflects broader inequalities in research funding, English-language publication infrastructure, and access to high-impact indexing platforms.

**Figure 10.**
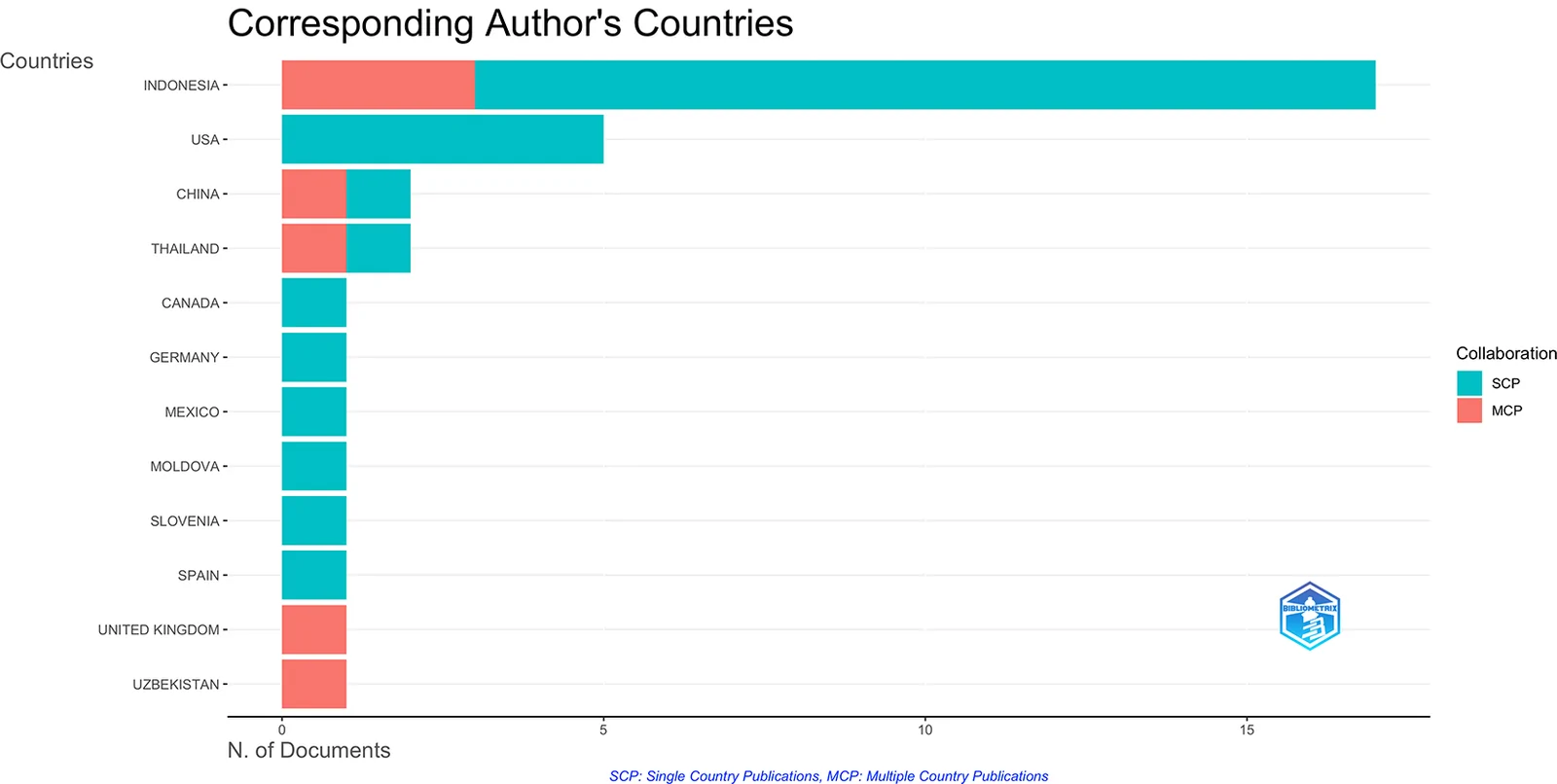
Corresponding authors’ countries.

Synthesizing across both figures, the co-authorship landscape of PBL/PjBL–EL research exhibits the characteristics of a field in early-to-middle internationalization: productive but regionally concentrated, selectively connected across national boundaries, and structurally dependent on a small number of high-centrality authors to bridge otherwise disconnected communities. The strategic importance of expanding this collaboration architecture cannot be overstated. Sustainability challenges—climate change, biodiversity loss, resource overconsumption—are inherently transnational and demand research frameworks that integrate diverse ecological, pedagogical, and sociocultural knowledge systems ( Wals & Benavot, 2017). SDG 17 (Partnerships for the Goals) and UNESCO’s ESD for 2030 roadmap both explicitly identify international research collaboration as a mechanism for accelerating sustainability education innovation; yet the present network suggests that this mandate has not yet translated into structurally equitable, globally integrated co-authorship practices ( Rieckmann, 2018). Future development of the field should prioritize North–South and South–South research partnerships, collaborative instrument validation studies, and multi-site longitudinal designs that embed diverse cultural and ecological contexts—moves that would simultaneously strengthen the methodological robustness of EL research and broaden its applicability to the full range of societies confronting sustainability transitions.

### 4.5. Content analysis of selected studies

Systematic content analysis was conducted across the 48 selected studies on the basis of article metadata, abstracts, keywords, institutional affiliations, learning outcomes, SDG alignment, and research methodologies. The findings are organized across five dimensions. To facilitate cross-category interpretation, percentages may sum to more than 100 in categories where studies adopt hybrid configurations (notably learning approach and research methodology); these overlaps are explicitly indicated where they occur.

4.5.1 Educational levels

Higher education is the dominant context, representing approximately 23 studies (48%), followed by secondary education (10 studies, 21%) and primary education (4 studies, 8%); the remainder involve teacher education or multi-level settings. This concentration reflects universities’ greater curricular autonomy, institutional SDG commitments, and structural capacity to integrate problem-based pedagogies ( Barth et al., 2022; Wiek et al., 2011). Engineering and science teacher education programs account for a disproportionate share of higher education studies, consistent with Rieckmann’s (2018) argument that teacher preparation constitutes a high-leverage multiplier for sustainability competency development. The under-representation of secondary education—constrained by rigid national curricula and standardized assessment pressures—and the near-absence of primary-level research (8%) constitute significant gaps, given that EL formation begins in early childhood ( Stevenson et al., 2013) and that SDG 4.7 mandates universal ESD across all educational levels.

4.5.2 Learning approaches

PjBL is the dominant pedagogy in 27 studies (56%) and PBL is dominant in 21 studies (44%), with 12 of these studies (25%) implementing hybrid PBL/PjBL configurations and therefore counted in both categories. Inquiry-based learning appears in three studies, collaborative learning in six, and technology-enhanced or blended designs in three—including an AI chatbot–flipped classroom module ( Wang & Li, 2026) and a digital eco-literacy platform ( Pratiwi, E. Y. R., et al., 2025). The dominance of PBL and PjBL reflects their theoretical alignment with constructivism ( Vygotsky, 1978), experiential learning ( Dewey, 1986), and ESD’s emphasis on authentic, student-centered engagement with real-world environmental problems ( Krajcik & Shin, 2014). A persistent gap, however, is that most studies document short-term outcome gains without examining whether PBL/PjBL-mediated learning produces sustained behavioral change or the perspective transformation that Mezirow (1991) and Sterling & Orr (2001) identify as the hallmark of genuine sustainability learning. Notably, only two studies explicitly anchor their pedagogical design in chemistry or environmental chemistry contexts, reinforcing the disciplinary asymmetry already detected in the keyword co-occurrence analysis.

4.5.3 Learning outcomes

EL is the most frequently targeted outcome (32 studies, 67%), followed by sustainability competencies (27 studies, 56%), ecological literacy (10 studies, 21%), problem-solving skills (6 studies), systems thinking (6 studies), and critical thinking (5 studies). Studies frequently target multiple outcomes simultaneously, which accounts for the cumulative percentage exceeding 100. A critical analytical finding is that EL is operationalized inconsistently across studies—variously as a knowledge construct, an awareness construct ( Wibowo et al., 2025), a competency construct ( Chien & Chien, 2025), a behavioral construct ( Ayerbe López & Perales Palacios, 2025), or a transformative construct ( Hedden et al., 2017; Lewinsohn et al., 2015). This conceptual fragmentation, combined with the widespread use of non-validated author-constructed measurement instruments ( Hollweg et al., 2011), severely limits cross-study comparability. Behavioral and civic action outcomes—central to ESD’s vision of environmentally responsible citizenship ( Roth, 1992)—remain markedly under-explored, suggesting that the field measures proximate cognitive outcomes rather than the distal transformative outcomes that sustainability education ultimately aims to produce.

4.5.4 SDG Integration

Explicit SDG alignment is surprisingly limited: only 12–15 studies directly invoke specific SDGs. Among those that do, SDG 12 (Responsible Consumption and Production) is most prevalent (approximately 8 studies), primarily through waste management and ecopreneurship contexts ( Armiati et al., 2026; Winarto et al., 2025); SDG 13 (Climate Action) appears in six studies; and SDG 4 (Quality Education, Target 4.7) is explicitly invoked in five studies as the ESD mandate ( Sposab et al., 2025; Torres-Rivera et al., 2025; Zirakian, 2026). SDGs 14 and 15 appear implicitly in biodiversity and coastal ecology studies. Most critically, the majority of studies treat the SDGs as thematic backdrop rather than as curricular architecture: problem scenarios may involve environmental issues, but instructional designs rarely systematically develop the five sustainability competencies—anticipatory, normative, strategic, collaborative, and self-awareness—that the Rieckmann (2017) framework prescribes. This superficial invocation risks reducing ESD to content delivery rather than transformative learning ( Jickling & Wals, 2008). Additionally, the social and economic dimensions of sustainability receive scant attention, with most studies addressing only the environmental pillar.

4.5.5 Research methodologies

Development and design-based research is the most prevalent methodological approach (approximately 19 studies, 40%), reflecting the field’s practical orientation toward creating and validating novel instructional materials and learning models. Qualitative designs appear in 17 studies (35%), quantitative and quasi-experimental designs in 15 studies (31%), and mixed-methods designs in nine studies (19%); these percentages exceed 100 because mixed-methods studies are counted under both qualitative and quantitative subcategories. Four methodological gaps are identified. First, longitudinal research is almost entirely absent: no study tracks EL development beyond a single academic term, leaving unanswered whether PBL/PjBL interventions produce lasting attitudinal and behavioral change. Second, instrument validation is insufficient; most studies rely on author-constructed measures without documented psychometric evidence, undermining meta-analytic synthesis. Third, in-depth qualitative exploration of transformative learning processes—phenomenological, narrative, or ethnographic—remains under-developed relative to the field’s transformative learning aspirations. Fourth, interdisciplinary research designs are rare, even when studies address inherently transdisciplinary environmental problems. Productive directions for future methodological innovation include design-based implementation research ( Fishman et al., 2013), learning analytics for near-real-time literacy trajectory mapping, and cross-cultural instrument validation studies capable of supporting rigorous international synthesis.

### 4.6. Thematic evolution and conceptual structure

The Sankey diagram ( Figure 11) and the multiple correspondence analysis thematic map ( Figure 12) jointly document a structural paradigmatic shift in the field rather than a mere expansion of topic coverage. In the 2011–2020 period, the dominant conceptual flows converge around ecological literacy and EL as foundational anchors, consistent with a knowledge-transmission model of environmental education. By the 2021–2026 period, these flows are substantially redistributed: project-based learning emerges as the dominant receiving node, absorbing intellectual energy previously concentrated in problem-based and knowledge-centered orientations, while students’ environmental engagement becomes a principal thematic output. This reorientation is not incidental—it reflects the field’s systemic alignment with UNESCO’s ESD framework and the broader competence-based turn in sustainability pedagogy ( Leicht et al., 2023; Wiek et al., 2015). The thematic map ( Figure 12) renders this structural logic spatially: motor themes—comprising problem-based learning, project-based learning, students, teaching, and curricula—occupy the high-centrality, high-density quadrant, confirming that active pedagogies have achieved theoretical maturation and serve as the organizing nucleus of contemporary research. Meanwhile, sustainability and higher education cluster in the upper-center zone with moderate density, indicating a trajectory toward motor status that has not yet fully consolidated. The authors interpret this configuration as evidence that the field is transitioning from a first-generation EL paradigm—rooted in ecological content knowledge—to a second-generation competence-based ESD paradigm in which pedagogical architecture, not knowledge transmission alone, constitutes the primary object of inquiry ( Cincera et al., 2024).

**Figure 11.**
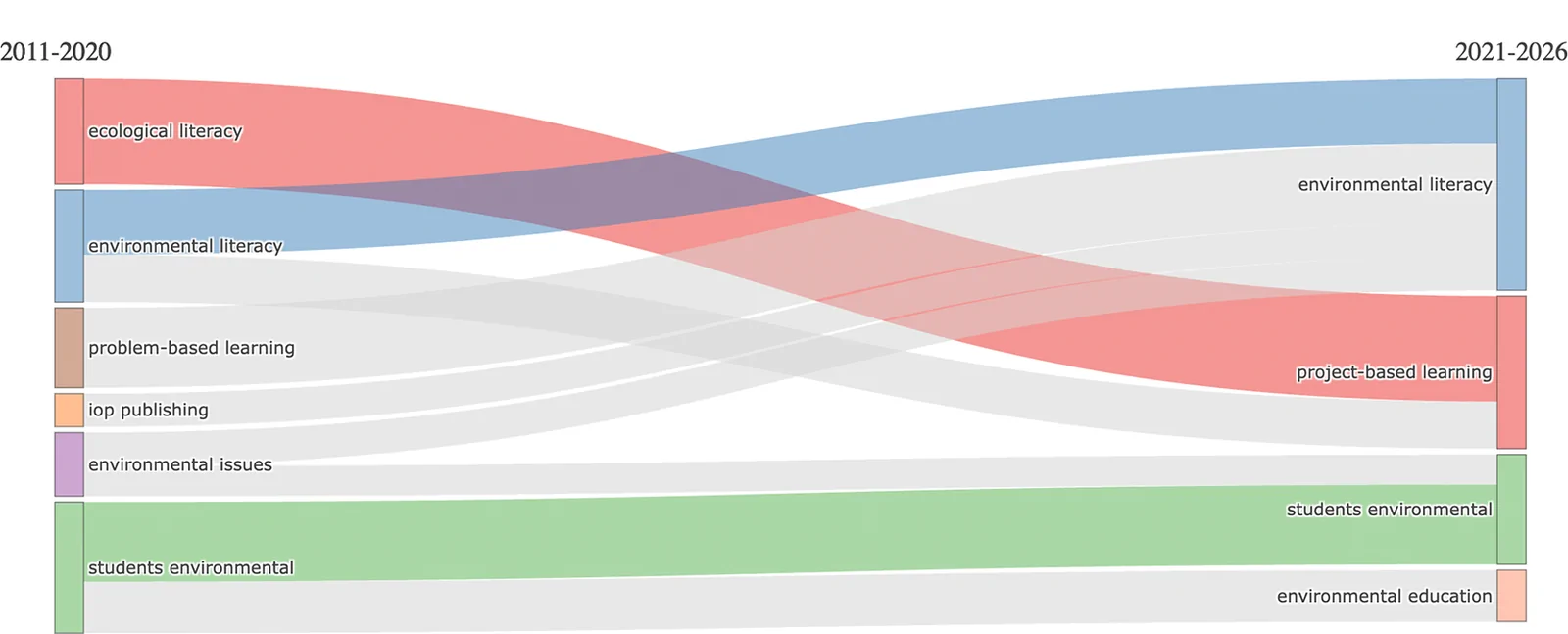
Sankey diagram of thematic evolution in environmental literacy research across two periods (2011–2020 and 2021–2026).

**Figure 12.**
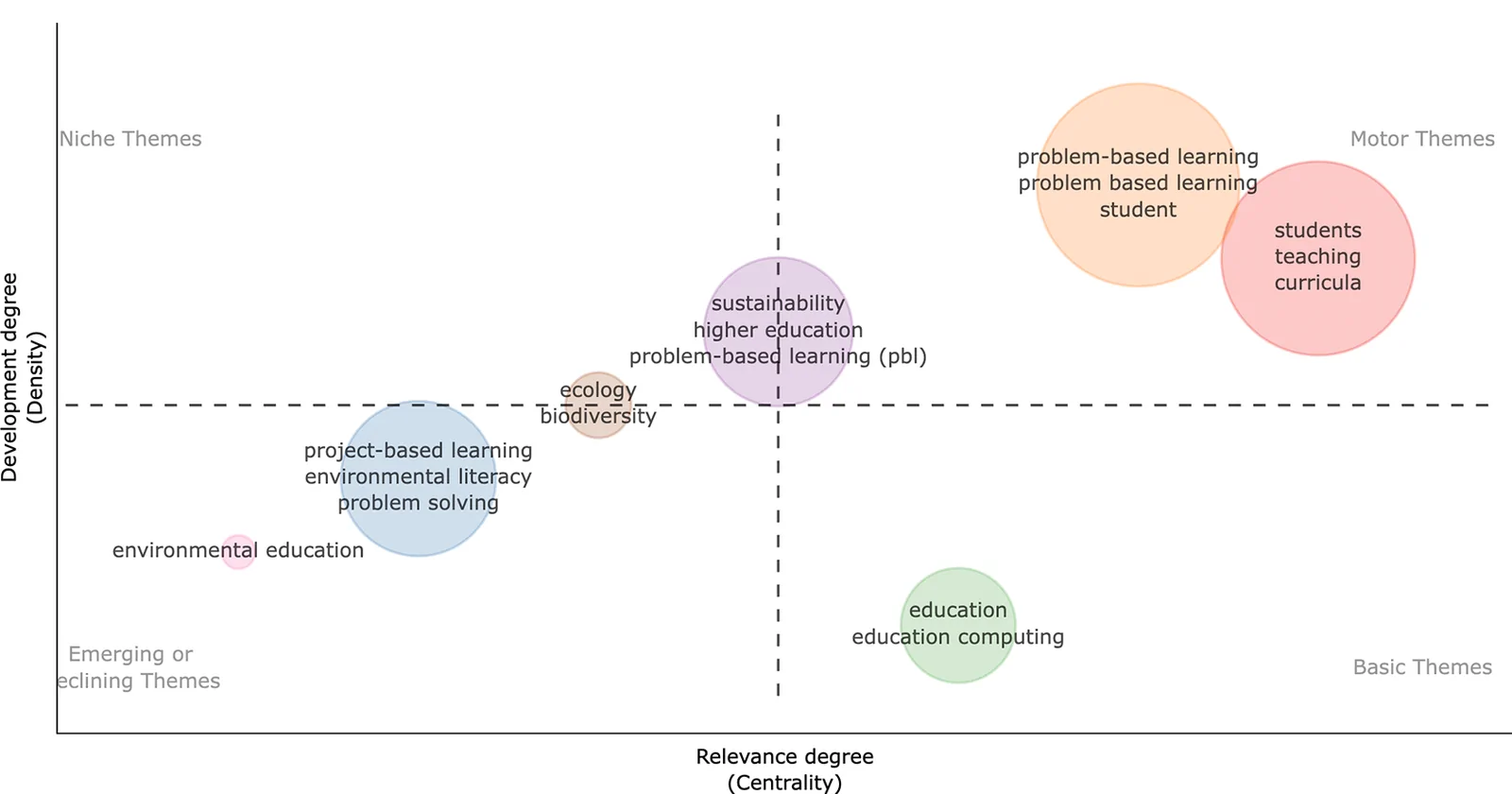
Thematic map.

The five keyword co-occurrence cluster networks ( Figures 13– 17) provide a granular topography of the field’s intellectual microstructure and reveal differentiated knowledge-production dynamics across each thematic zone. Cluster 1 ( Figure 13, orange), anchored in “problem-based learning,” “student,” and “sustainable development,” is the most densely interconnected network, displaying strong internal cohesion among pedagogical process terms (literacy, human, procedures, artificial intelligence) and a direct connection to the sustainable development agenda—suggesting that PBL is increasingly theorized as a scalable mechanism for sustainability competence, not merely a classroom technique. Cluster 2 ( Figure 14, red), centered on “students,” “teaching,” and “curricula,” forms an elongated network bridging environmental impact assessment, climate change, and engineering curricula, which the authors read as evidence that EL is being institutionally embedded in disciplinary degree programs, particularly in engineering and technical education ( Brundiers et al., 2021; Leal Filho et al., 2021). Cluster 3 ( Figure 15, purple) reveals “sustainability” and “higher education” as the pivot of a loosely coupled network incorporating design thinking, human-centered design, interdisciplinary pedagogy, and STEAM—reflecting the field’s growing engagement with transformative design-based pedagogies that challenge the conventional PBL/PjBL binary. Cluster 4 ( Figure 16, blue), organized around “project-based learning” and “environmental literacy,” constitutes the most methodologically coherent cluster, integrating pro-environmental attitude, environmental awareness, socio-scientific issues, and local wisdom—signaling a contextual and place-based pedagogical turn in which PjBL is deployed within culturally situated, community-level environmental problems ( Fauzi & Wijarini, 2024). Cluster 5 ( Figure 17, green), anchored in “education” and “education computing,” connects Industry 4.0 technologies, greenhouse gas emissions, and pedagogical strategies, indicating that digital and computational tools are beginning to enter EL research as both instructional media and analytical frameworks—a convergence that remains nascent but carries significant implications for scalable ESD delivery.

**Figure 13.**
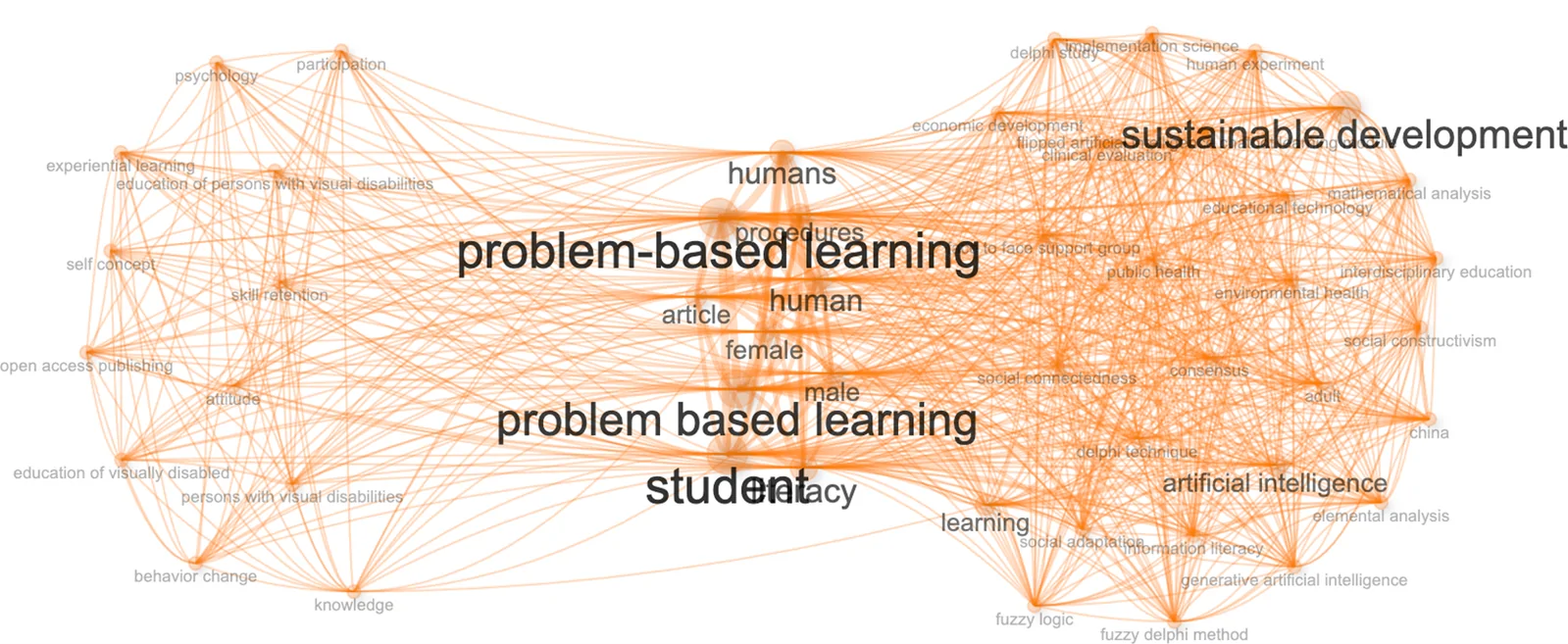
Keyword Co-occurrence Network for Cluster 1: Problem-Based Learning and Sustainable Development.

**Figure 14.**
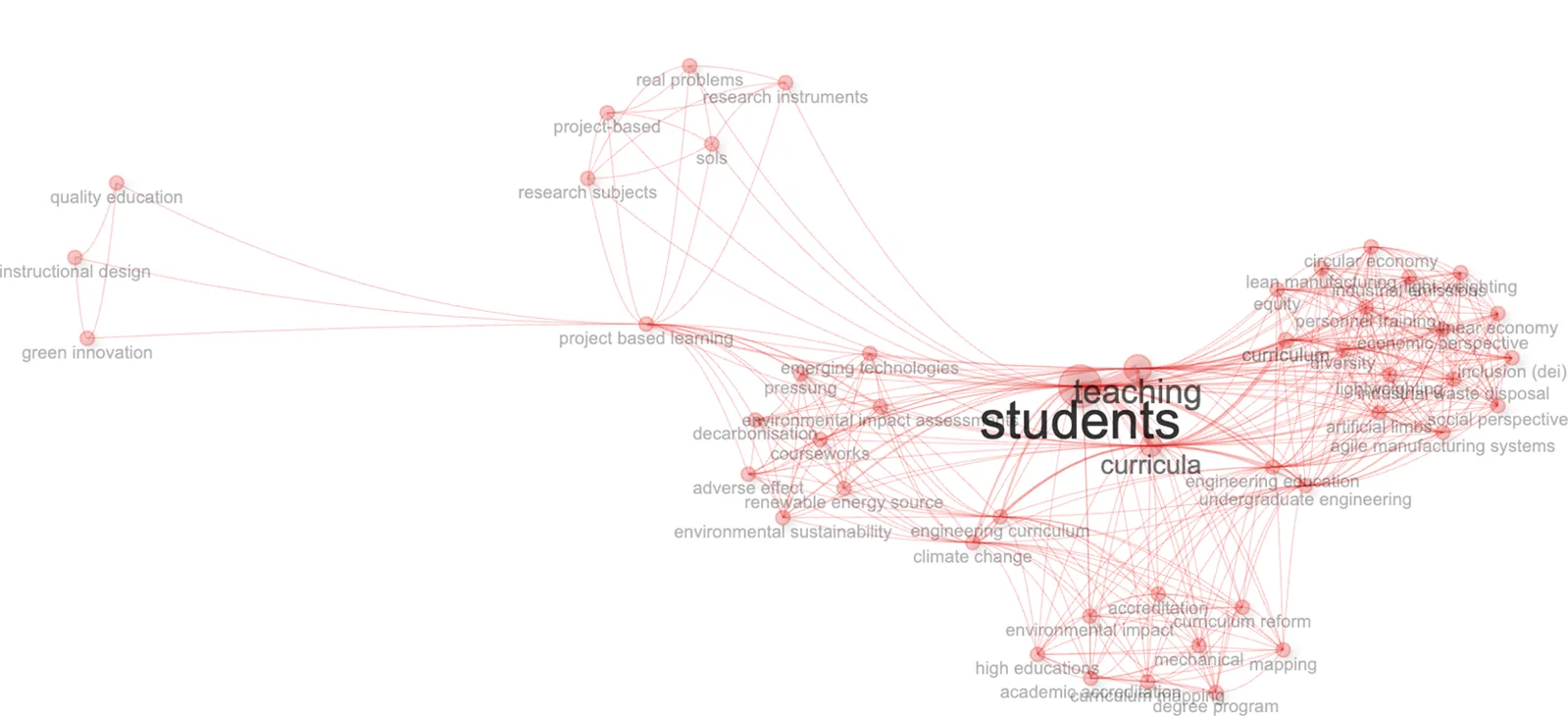
Keyword Co-occurrence Network for Cluster 2: Students, Teaching, and Curricula in Environmental Education.

**Figure 15.**
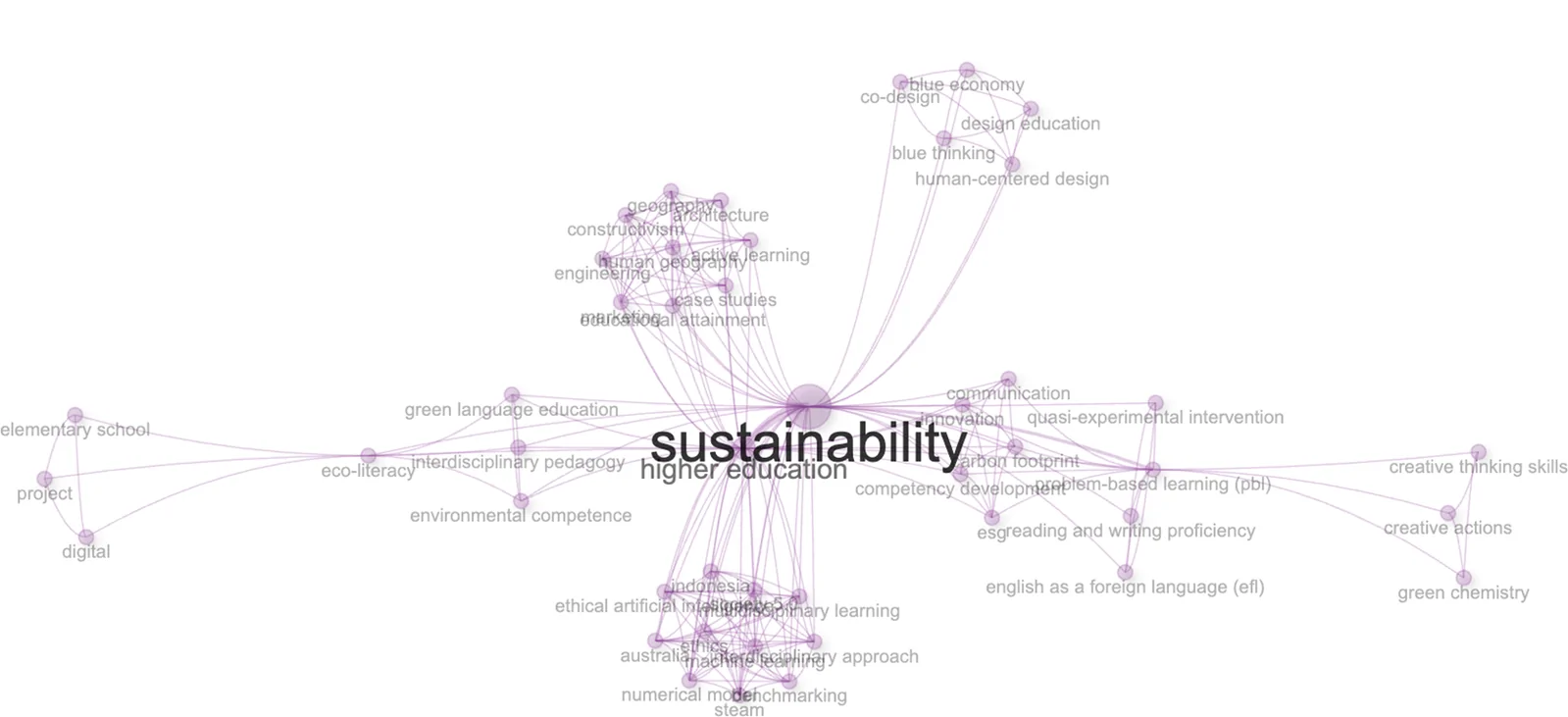
Keyword Co-occurrence Network for Cluster 3: Sustainability and Higher Education Pedagogy.

**Figure 16.**
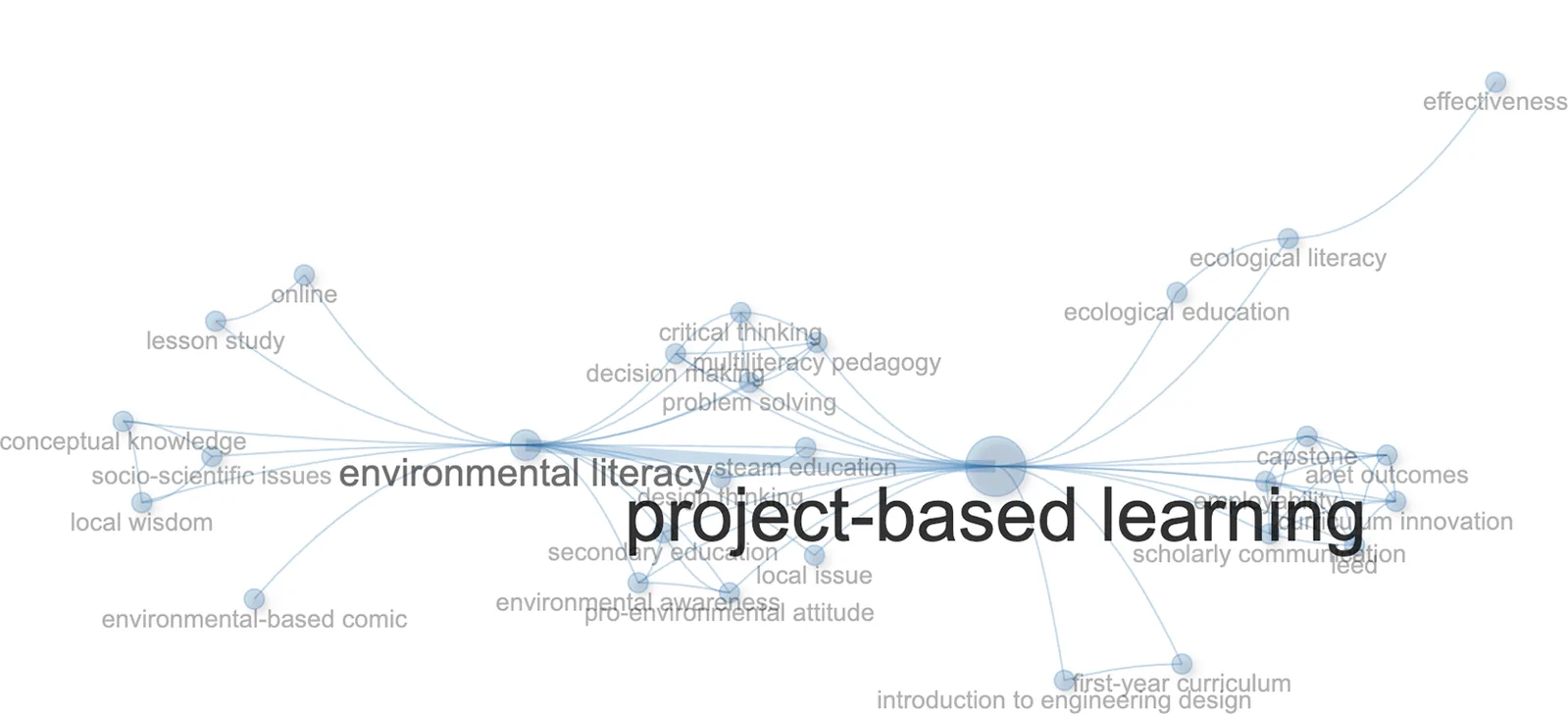
Keyword Co-occurrence Network for Cluster 4: Project-Based Learning and Environmental Literacy.

**Figure 17.**
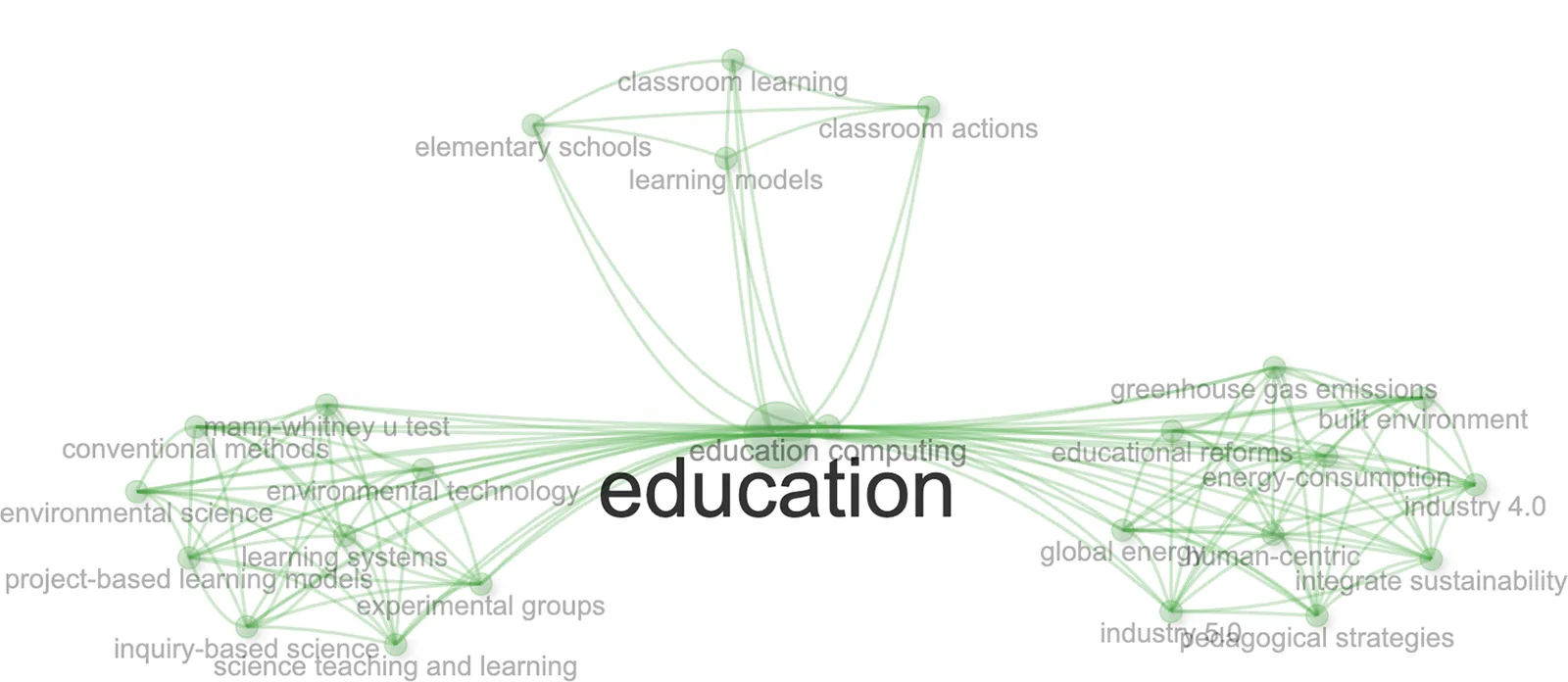
Keyword Co-occurrence Network for Cluster 5: Education and Technology Integration.

Synthesizing across all seven visualizations, the authors argue that the most consequential finding is not any single thematic shift, but rather the structural decoupling between the field’s motor core and its emerging peripheral zones. The motor themes ( Figures 12– 14) are tightly internally integrated but show limited connective tissue to the sustainability-design cluster ( Figure 15) and the technology-integration cluster ( Figure 17). This topological gap indicates that the field has produced two relatively insular knowledge streams: one pedagogically mature but methodologically conservative, and one methodologically innovative but empirically thin. The dominance of project-based learning as the receiving node in Figure 11 is epistemologically significant: it positions PjBL not as a teaching tool subordinate to content, but as the generative mechanism through which environmental knowledge, sustainability competencies, and disciplinary curricula are brought into productive alignment. However, the relative peripherality of eco-literacy, innovation, and digital learning in the emerging/declining quadrant of Figure 12—combined with their sparse co-occurrence density in Figure 17—suggests that the field has not yet theoretically integrated the affordances of technology-mediated learning within its active-pedagogy framework. The authors therefore contend that the next productive research frontier lies not in further consolidating motor themes, but in building cross-cluster bridging research that connects the PBL/PjBL core with design-based, AI-integrated, and place-based pedagogies—precisely the intersections that current thematic and co-occurrence structures identify as structurally present but conceptually underdeveloped ( Wiek et al., 2015).

### 4.7. Research gaps and future directions

The bibliometric mapping and systematic content analysis collectively reveal that, despite the rapidly expanding scholarly interest in problem-based and project-based learning for EL, the field continues to exhibit conceptual, methodological, and contextual asymmetries that constrain its transformative potential within sustainability-oriented science education. A particularly conspicuous gap concerns the under-representation of chemistry and environmental chemistry education, where PBL and PjBL applications remain peripheral despite the discipline’s strategic relevance to green chemistry, climate science, and the molecular dimensions of EL. Within the present corpus, only two studies engaged chemistry as a disciplinary anchor, despite the centrality of chemical literacy to scientifically informed environmental decision-making ( Mahaffy et al., 2019). Equally under-developed is the systematic integration of PBL pedagogies with the SDGs and EL frameworks; existing studies tend to address these constructs in parallel rather than weaving them into coherent pedagogical architectures capable of cultivating sustainability competencies. The relative scarcity of psychometrically robust, contextually validated instruments for assessing EL further compounds this fragmentation, as scholarship in the field continues to rely on heterogeneous and often non-replicable measurement approaches. Compounding these limitations is the dominance of short-term, quasi-experimental designs over longitudinal and transformative learning studies, which restricts the field’s capacity to capture deep and durable shifts in learners’ values, dispositions, and pro-environmental behaviors. Insufficient interdisciplinary integration—particularly across the natural sciences, social sciences, and humanities—has further produced a body of literature that struggles to address the inherently complex, wicked character of sustainability problems, while limited engagement with systems thinking and key sustainability competencies has weakened alignment with global ESD agendas. Future research should therefore prioritize the design of longitudinal, mixed-methods studies that trace transformative learning trajectories within authentic socio-ecological contexts; develop and validate culturally responsive EL instruments; and deliberately embed climate education, systems thinking, and the eight UNESCO key sustainability competencies into PBL and PjBL ecosystems. Strategic expansion into chemistry and environmental chemistry education is particularly imperative, as it represents an underutilized epistemic frontier for advancing molecular-level EL and connecting laboratory-based inquiry to planetary sustainability concerns. Strengthening SDG-oriented science education frameworks will ultimately require intentional convergence among pedagogical innovation, interdisciplinary inquiry, and policy-relevant educational design—an integrative trajectory that repositions PBL and PjBL not merely as instructional methods but as enabling frameworks for cultivating ecologically literate, future-ready citizens.

## 5. Conclusion

This study advances a thematically focused scientific mapping of problem-based and project-based learning research in EL education by integrating bibliometric performance analysis, science mapping, and systematic content analysis within a unified analytical architecture. Within the bounded but conceptually coherent corpus of 48 Scopus-indexed articles published between 2011 and 2026, three findings stand out. First, the field has transitioned from a decade of intellectual latency into a phase of accelerated post-2021 expansion, driven by the convergence of the ESD for 2030 framework, the consolidation of sustainability competence frameworks, and intensified institutional engagement with the SDGs. Second, the intellectual structure of the field is organized around a stable PBL/PjBL–EL core that is increasingly articulated with sustainability-oriented curriculum reform, climate education, and eco-literacy innovation; yet the conceptual vocabulary connecting these clusters to systems thinking, transformative learning, and environmental citizenship remains under-developed. Third, the field’s geopolitical configuration is shifting, with Indonesia and a small set of Southeast Asian institutions emerging as major contributors alongside established North American and European centers, but with persistent under-representation of Africa, Latin America, and South Asia.

Theoretically, the integration of bibliometric mapping with systematic content analysis offers a replicable analytical architecture in which macro-structural and substantive findings can be triangulated. Practically, the study provides curriculum designers, sustainability officers, and teacher educators with a defensible evidence base for embedding PBL and PjBL as core pedagogies of EL and sustainability competence. The strategic implication is that PBL and PjBL should be conceptualized not as discrete instructional techniques but as integrative pedagogies capable of operationalizing the ESD agenda within disciplinary contexts—including, critically, chemistry and environmental chemistry education, where their potential remains conspicuously under-explored. As sustainability-oriented science education enters an era defined by climate urgency, technological mediation, and the imperative for transdisciplinary collaboration, the field’s continued maturation will depend on its capacity to integrate longitudinal evidence, validated instruments, transversal conceptual vocabulary, and equitable Global-North–Global-South partnerships. PBL and PjBL, properly theorized and rigorously implemented, can serve as powerful enabling pedagogies for the next generation of environmentally literate, sustainability-competent learners.

## 6. Limitations and future research

Several methodological and contextual limitations bound the present study and delineate productive directions for future inquiry. First, the exclusive reliance on Scopus, while justified by its indexing rigor and metadata consistency, restricts the corpus relative to multi-database designs that incorporate Web of Science, Dimensions, and ERIC. Second, the English-language restriction under-represents non-Anglophone scholarship, particularly from Latin America, Francophone Africa, and parts of Asia where culturally embedded environmental education traditions remain analytically rich. Third, the exclusion of conference proceedings, book chapters, and grey literature limits the visibility of emergent practitioner-oriented work. Fourth, the modest corpus size (n = 48) constrains the statistical stability of cluster solutions and life-cycle modeling; small-corpus bibliometric outputs should therefore be read as descriptive rather than predictive. Fifth, although systematic content analysis was applied to triangulate the bibliometric findings and inter-coder reliability achieved substantial agreement (κ = 0.81–0.89), the codebook itself remains theoretically contingent and should be refined in future work as the field continues to mature. Sixth, the keyword strategy, while deliberately balanced between recall and precision, may have introduced selection asymmetries that shape thematic clustering.

Future research should therefore advance toward methodological pluralism: multi-database bibliometric designs; multilingual and decolonial keyword strategies that capture indigenous environmental knowledge systems and Global South scholarship; mixed bibliometric–scoping–meta-synthesis designs; longitudinal and design-based research tracing EL trajectories beyond a single semester; and validated, cross-culturally adapted EL instruments capable of supporting rigorous international synthesis. Particular attention should be directed to chemistry and environmental chemistry education, where the integration of green chemistry, planetary boundaries, and laboratory-based inquiry within PBL/PjBL architectures represents an under-exploited frontier for advancing molecular-level EL. Strengthening collaborative infrastructures across the Global North and Global South, supporting open-access dissemination, and embedding EL within institutional sustainability strategies are not auxiliary tasks but central conditions for advancing transformative environmental learning at a planetary scale.

## Data Availability

The bibliographic dataset, completed PRISMA 2020 checklist, and PRISMA 2020 flow diagram supporting the findings of this systematic review and bibliometric study are openly available in the Open Science Framework (OSF) repository under a Creative Commons Zero (CC0 1.0 Universal) public domain dedication. The repository record is accessible at https://doi.org/10.17605/OSF.IO/XBDVA. The repository contains the following materials: (1) the Scopus bibliographic export (BibTeX and xlsx formats) comprising all 48 articles included in the final analytical corpus; (2) the completed PRISMA 2020 checklist and (3) the PRISMA 2020 flow diagram documenting the identification, screening, eligibility assessment, and inclusion stages of the literature search. Formal citation: Mellyzar, Nahadi, Hernani, Supriatna, A., Oktariani, Marhami, & Nabuasa, D. A. (2026). PRISMA Checklist and Flow Diagram – Scientific Mapping of PBL/PjBL and Environmental Literacy Education. Open Science Framework (OSF). https://doi.org/10.17605/OSF.IO/XBDVA. Licensed under CC0 1.0 Universal.
