# Supplementary material for: Scientific Mapping of Problem-Based and Project-Based Learning for Environmental Literacy Education: A Bibliometric and Content Analysis
Source: F1000Res. 2026 Nov 12;15:1323. Originally published 2026 Aug 7. [Version 2] doi: 10.12688/f1000research.182697.2 (PMC13768705; doi:10.12688/f1000research.182697.2)
Supplement: Peer review history

# Reviewer report: Reviewer response for version 2

**DOI:** 10.5256/f1000research.213663.r541173

**Published:** 2026-11-13

**Authors:** Sri Nurhayati

**Affiliations:** 1 IKIP Siliwangi, Cimahi, West Java, Indonesia

the authors have made the revisions following the comments

Are the rationale for, and objectives of, the Systematic Review clearly stated?

Yes

Is the statistical analysis and its interpretation appropriate?

Not applicable

If this is a Living Systematic Review, is the ‘living’ method appropriate and is the search schedule clearly defined and justified? (‘Living Systematic Review’ or a variation of this term should be included in the title.)

Not applicable

Are sufficient details of the methods and analysis provided to allow replication by others?

Yes

Are the conclusions drawn adequately supported by the results presented in the review?

Yes

Reviewer Expertise:

Problem based Learningproject based learning

I confirm that I have read this submission and believe that I have an appropriate level of expertise to confirm that it is of an acceptable scientific standard.

# Reviewer report: Reviewer response for version 1

**DOI:** 10.5256/f1000research.201661.r523056

**Published:** 2026-10-01

**Authors:** Sri Nurhayati

**Affiliations:** 1 IKIP Siliwangi, Cimahi, West Java, Indonesia

The manuscript presents a timely and potentially valuable bibliometric and systematic content analysis of Problem-Based Learning (PBL), Project-Based Learning (PjBL), and environmental literacy (EL). The integration of science mapping and content analysis is appropriate for identifying both the structural development and substantive characteristics of this emerging field. The manuscript is generally well organized and provides several interesting findings, particularly the rapid post-2021 growth of the field, the dominance of higher education, the limited representation of chemistry and environmental chemistry education, and the uneven geographical distribution of scholarship. The manuscript also demonstrates good awareness of its methodological limitations, including the exclusive use of Scopus, English-language restriction, and the relatively small corpus of 48 studies.

 For revision, I encourage the authors to strengthen the methodological transparency of the systematic content analysis. Although inter-coder reliability is reported as substantial (κ = 0.81–0.89), the manuscript would benefit from explicitly explaining how many studies were independently double-coded, which coding categories were assessed, when reliability was calculated, and how disagreements were resolved. The operationalization of SDG alignment should also be clarified, particularly whether alignment was explicitly reported by the original studies or assigned retrospectively by the authors.

 The Discussion would benefit from substantially deeper critical interpretation rather than primarily restating bibliometric patterns. For example, the post-2021 increase is currently linked to ESD 2030, sustainability competencies, and SDG engagement; however, these should be presented as plausible contextual explanations rather than causal drivers unless directly supported by the analysis. Similarly, claims concerning Global South representation should explicitly acknowledge that the evidence reflects Scopus-indexed, English-language scholarship rather than the entire global research landscape.

 Finally, the authors should sharpen the theoretical implications by explaining what the mapping reveals about the relationship between PBL/PjBL, EL, and transformative sustainability education. The conclusion should distinguish clearly between what the bibliometric evidence demonstrates and what the authors propose as future directions. These focused revisions would substantially strengthen the manuscript while remaining consistent with a Minor Revision decision.

Are the rationale for, and objectives of, the Systematic Review clearly stated?

Yes

Is the statistical analysis and its interpretation appropriate?

Not applicable

If this is a Living Systematic Review, is the ‘living’ method appropriate and is the search schedule clearly defined and justified? (‘Living Systematic Review’ or a variation of this term should be included in the title.)

Not applicable

Are sufficient details of the methods and analysis provided to allow replication by others?

Yes

Are the conclusions drawn adequately supported by the results presented in the review?

Yes

Reviewer Expertise:

Problem based Learningproject based learning

I confirm that I have read this submission and believe that I have an appropriate level of expertise to confirm that it is of an acceptable scientific standard, however I have significant reservations, as outlined above.

# Response

**Published:** 2026-10-29

**Authors:** Mellyzar Mellyzar

**Affiliations:** Science Education, Universitas Pendidikan Indonesia, Bandung, West Java, Indonesia

**Response to Reviewer**

 Dear Reviewer,

 We sincerely thank you for your constructive and encouraging assessment of our manuscript. We appreciate your recognition of the relevance of the study and the value of integrating bibliometric science mapping with systematic content analysis. Your comments have provided valuable guidance for improving the methodological transparency, analytical depth, and theoretical contribution of the manuscript. We have carefully considered your recommendations and revised the manuscript accordingly. Our responses are provided below.

**Comment 1. Methodological transparency and inter-coder reliability**

 The reviewer recommended clarifying the number of independently double-coded studies, coding categories, timing of reliability assessment, and procedures for resolving disagreements.

**Response:**

 Thank you for this important recommendation. We have substantially revised Section 3.7 to clarify the content analysis procedures. The revised manuscript specifies that 44 full-text records underwent two separate AI-assisted coding runs using a structured codebook covering five analytical categories: educational level, learning approach, learning outcomes, SDG alignment, and research methodology. Cohen's kappa was calculated from the initial coding outputs before disagreements were resolved. Agreement across the five categories ranged from κ = 0.87 to κ = 0.97, while eligibility decisions showed complete agreement (κ = 1.00).

 We have also clarified the role of researcher verification. All coding outputs were manually examined against the original full-text articles by two authors. A total of 26 discrepancies across 17 studies were reviewed and resolved, with the decisions and supporting rationales documented in a disagreement log. One additional study (S35) was subsequently coded and verified but was excluded from the reliability calculation because it had not undergone independent double coding. The coding framework and reliability results are presented in Table 2.

**Comment 2. Operationalization of SDG alignment**

 The reviewer requested clarification of whether SDG alignment was explicitly reported in the original studies or assigned retrospectively by the authors.

**Response:**

 We agree that distinguishing explicit SDG integration from retrospectively identified thematic alignment is essential for methodological clarity. Accordingly, we have refined the coding definitions and revised the relevant methodological and analytical sections to differentiate SDGs explicitly named by the original authors from SDGs assigned through thematic interpretation during content analysis.

 The revised findings distinguish intentional SDG integration from broader thematic correspondence. This clarification prevents the overinterpretation of sustainability-related learning activities as evidence that the original studies explicitly incorporated the SDGs into their instructional design.

**Comment 3. Critical interpretation of publication trends**

 The reviewer suggested strengthening the Discussion and avoiding unsupported causal interpretations of the increase in publications after 2021.

**Response:**

 Thank you for this valuable observation. We have revised the Discussion to move beyond the descriptive presentation of bibliometric patterns and provide a more critical interpretation of the findings.

 In particular, we now describe the post-2021 increase in publication output as coinciding with broader developments in sustainability education, including the ESD for 2030 framework and growing attention to sustainability competencies. We acknowledge that these developments provide plausible contextual explanations rather than demonstrated causal relationships. The revised discussion therefore more clearly distinguishes observed publication trends from their possible interpretations.

**Comment 4. Geographical representation and limitations**

 The reviewer recommended qualifying claims about Global South representation to reflect the geographical and linguistic limitations of the database.

**Response:**

 We appreciate this important recommendation. We have revised the interpretation of geographical and institutional patterns to clarify that the findings reflect publications indexed in Scopus and written in English, rather than the entire global research landscape.

 The revised discussion and limitations acknowledge that studies published in regional journals, other databases, and non-English outlets may be underrepresented. Consequently, conclusions concerning geographical participation and Global South representation are now framed within the coverage and selection boundaries of the analyzed corpus.

**Comment 5. Theoretical implications and conclusions**

 The reviewer recommended strengthening the theoretical implications concerning PBL/PjBL, environmental literacy, and transformative sustainability education, and distinguishing empirical findings from future research proposals.

**Response:**

 Thank you for this insightful recommendation. We have strengthened the theoretical discussion by examining how PBL and PjBL are positioned in relation to environmental literacy and transformative sustainability education. In particular, the revised analysis distinguishes the pedagogical potential of these approaches from evidence of measured environmental literacy outcomes and demonstrated transformative learning.

 The conclusion has also been refined to distinguish findings directly supported by the bibliometric and content analyses from recommendations for future research. These recommendations include longitudinal investigations, more consistent and validated environmental literacy assessments, explicit SDG-oriented instructional design, and greater integration of chemistry and environmental chemistry education.

 We believe these revisions provide a clearer account of the study's empirical contributions and their implications for sustainability-oriented science education.

**Closing statement**

 We sincerely appreciate the reviewer's thoughtful and constructive feedback. The revisions have strengthened the methodological transparency, analytical consistency, theoretical interpretation, and overall clarity of the manuscript. We hope that the revised version satisfactorily addresses the concerns raised.

 Kind regards,

 The Authors

# Reviewer report: Reviewer response for version 1

**DOI:** 10.5256/f1000research.201661.r523055

**Published:** 2026-09-22

**Authors:** Nikolaos Pellas

**Affiliations:** 1 Aristotle University of Thessaloniki, Thessaloniki, Greece

Major comments and specific questions

**1. The search strategy needs substantially more detail.**

 The manuscript states that a Boolean query was used, but the actual search string, fields searched, date of the search, and inclusion/exclusion criteria are not presented in the excerpt. This is critical for reproducibility.

**Question:** *Could the authors provide the complete Boolean search string, the Scopus search fields, search date, date range, and all inclusion/exclusion criteria?*

 In particular, how were **PBL**, **PjBL**, **environmental literacy**, **environmental education**, **sustainability education**, and **ESD** operationalised in the search?

**2. The corpus of only 48 studies raises concerns about the robustness of bibliometric mapping.**

 The authors acknowledge this limitation, but the manuscript nevertheless makes relatively strong claims about “intellectual structure,” “thematic evolution,” and “geopolitical configuration.”

**Question:** *What minimum-occurrence thresholds and clustering parameters were used in VOSviewer and Bibliometrix, and how sensitive were the resulting clusters to changes in these parameters?*

 With **n = 48**, it would be useful to demonstrate that the identified four clusters are not artefacts of particular parameter choices.

**3. There appears to be a discrepancy between the reported thematic clusters and the reported findings.**

 The abstract identifies **four thematic clusters**, whereas the conclusion describes a “stable PBL/PjBL–EL core” and several additional conceptual areas. The relationship between these categories is not entirely clear.

**Question:** *Could the authors explicitly show how the four clusters were derived and how each cluster was interpreted conceptually?*

 A table containing cluster label, dominant keywords, number of documents, representative studies, and interpretation would considerably improve transparency.

**4. Clarify the distinction between PBL and PjBL.**

 The manuscript frequently combines problem-based learning and project-based learning as “PBL/PjBL.” However, these are conceptually distinct pedagogical approaches.

**Question:** *How did the review distinguish problem-based learning from project-based learning during screening and content analysis?*

 Were they analysed separately before being combined, and are there meaningful differences in their relationships with environmental literacy or sustainability competencies?

 This distinction is particularly important because otherwise the synthesis risks treating two related but non-equivalent pedagogies as a single intervention category.

**5. The definition of environmental literacy requires greater conceptual precision.**

 “Environmental literacy” appears to encompass knowledge, skills, attitudes, values, systems thinking, and pro-environmental behaviour. These dimensions are not necessarily interchangeable.

**Question:** *What definition of environmental literacy guided study inclusion and coding, and which dimensions of EL were actually represented in the 48 studies?*

 I would encourage the authors to report whether studies measured **knowledge, affective dispositions, environmental attitudes, competencies, behaviours, systems thinking, or combinations thereof**.

**6. The claim that PBL/PjBL are “enabling pedagogies” may go beyond the evidence.**

 A bibliometric/content analysis can identify patterns in the literature, but it cannot itself establish that PBL or PjBL produces transformative environmental learning.

 For example, the conclusion states that PBL and PjBL “can serve as powerful enabling pedagogies.” This is stronger than a descriptive bibliometric finding.

**Question:** *Which findings from the reviewed studies justify moving from mapping the literature to making claims about the effectiveness or transformative potential of PBL/PjBL?*

 The authors should distinguish clearly between **what the bibliometric analysis demonstrates**, **what individual empirical studies report**, and **the authors' interpretation or recommendation**.

 Methodological issues

**7. Clarify the relationship between PRISMA 2020 and bibliometric review methodology.**

 The manuscript states that studies were “screened in accordance with the PRISMA 2020 framework.”

**Question:** *Was PRISMA 2020 used only to report identification and screening, or was a specific systematic-review methodology also followed for eligibility and synthesis?*

 If this is primarily a bibliometric review, the authors should explain how PRISMA was adapted to the bibliometric design rather than implying that PRISMA itself constitutes the review methodology.

**8. Why was Scopus the sole database?**

 The limitations acknowledge this, but the issue has implications beyond a generic limitation because the review makes claims about the **global intellectual structure** and **geopolitical representation**.

**Question:** *To what extent can conclusions about Global South representation be interpreted as characteristics of the field rather than characteristics of Scopus-indexed, English-language scholarship?*

 This distinction is particularly important given the stated concern about Africa, Latin America, and South Asia.

**9. The geopolitical analysis needs methodological clarification.**

 The manuscript states that Indonesian and North American institutional clusters dominate and that Africa, Latin America, and South Asia are underrepresented.

**Question:** *How was geographical representation operationalised—by author affiliation, corresponding-author affiliation, institutional location, country of publication, or another criterion?*

 Also, “institutional clusters” and “countries” should not be treated as equivalent units of analysis.

**10. The interpretation of post-2021 growth needs greater caution.**

 The manuscript attributes the accelerated trajectory after 2021 to the ESD for 2030 framework, sustainability competence frameworks, and intensified institutional engagement with SDGs.

 However, an increase in publications after 2021 does not by itself demonstrate that these developments caused the increase.

**Question:** *What evidence supports the proposed explanation for the post-2021 increase, as opposed to simply documenting a temporal increase in publication output?*

 The authors might present these factors as **possible contextual explanations** rather than causal drivers unless the study actually analyses them.

**11. Clarify how SDG alignment was determined.**

 The statement that SDGs 4, 12, and 13 are “the most invoked goals” requires methodological explanation.

**Questions:**

- Were SDGs explicitly mentioned by the authors of the included papers?
- Were SDGs assigned by the present authors based on study content?
- Could a single study be coded against multiple SDGs?
- Was there an explicit coding framework for SDG alignment?

This is important because **explicit SDG invocation** and **post hoc conceptual alignment with an SDG** are different forms of evidence.

**12. The content-analysis reliability is reported, but the coding process needs more information.**

 Reporting κ = 0.81–0.89 is useful, but readers need to know how this was obtained.

**Question:** *How many studies were independently double-coded, which coding dimensions were included in the reliability calculation, and was reliability calculated before or after reconciliation between coders?*

 Also, were disagreements resolved through discussion, adjudication, or a predefined procedure?

Are the rationale for, and objectives of, the Systematic Review clearly stated?

Yes

Is the statistical analysis and its interpretation appropriate?

Not applicable

If this is a Living Systematic Review, is the ‘living’ method appropriate and is the search schedule clearly defined and justified? (‘Living Systematic Review’ or a variation of this term should be included in the title.)

Not applicable

Are sufficient details of the methods and analysis provided to allow replication by others?

Yes

Are the conclusions drawn adequately supported by the results presented in the review?

Yes

Reviewer Expertise:

AI in education

I confirm that I have read this submission and believe that I have an appropriate level of expertise to confirm that it is of an acceptable scientific standard.
